# Type 2 diabetes linked *FTO* gene variant rs8050136 is significantly associated with gravidity in gestational diabetes in a sample of Bangladeshi women: Meta-analysis and case-control study

**DOI:** 10.1371/journal.pone.0288318

**Published:** 2023-11-30

**Authors:** U. S. Mahzabin Amin, Tahia Anan Rahman, Mashfiqul Hasan, Tania Tofail, Muhammad Abul Hasanat, Zeba I. Seraj, Md Salimullah

**Affiliations:** 1 Molecular Biotechnology Division, National Institute of Biotechnology (NIB), Savar, Dhaka, Bangladesh; 2 Department of Endocrinology, Bangabandhu Sheikh Mujib Medical University (BSMMU), Dhaka, Bangladesh; 3 Department of Biochemistry and Molecular Biology, University of Dhaka, Dhaka, Bangladesh; Shaheed Rajaei Hospital: Rajaie Cardiovascular Medical and Research Center, ISLAMIC REPUBLIC OF IRAN

## Abstract

**Objective:**

Gestational diabetes mellitus (GDM) is a growing public health concern that has not been extensively studied. Numerous studies have indicated that a variant (rs8050136) of the fat mass-associated gene, *FTO*, is associated with both GDM and Type 2 diabetes mellitus(T2DM). We conducted a meta-analysis on the association between the *FTO* single nucleotide polymorphism (SNP) rs8050136 and T2DM, followed by a case-control study on the association of the said SNP and GDM in a sample of Bangladeshi women.

**Method:**

A total of 25 studies were selected after exploring various databases and search engines, which were assessed using the Newcastle-Ottawa Scale (NOS). The MetaGenyo web tool was used to conduct this meta-analysis. A case-control study was performed on 218 GDM patients and 284 controls to observe any association between *FTO* rs8050136 and GDM. Genotyping was performed using the tetra-primer amplification refractory mutation system-polymerase chain reaction (T-ARMS) method, and statistical analyses were performed using various statistical softwares.

**Results:**

In the meta-analysis 26231 cases and 43839 controls were examined. Pooled association analyses revealed a statistically significant relationship between the *FTO* rs8050136 polymorphism and an elevated risk of T2DM under all genetic models (*P<*0.05). In the case-control study, synergistic analyses of the SNP and gravida with GDM revealed a significant (*P*<0.01) association with an increase in odds by 1.6 to 2.4 folds in multigravida and decrease in odds by 2 folds in primigravida. A positive family history of diabetes and the minor allele of this SNP collectively increased the risk of developing GDM by many-fold (1.8 to 2.7 folds). However, after accounting for family history of diabetes and gravidity, analyses showed no significant association with GDM.

**Conclusion:**

Our meta-analysis revealed a significant association between SNP rs8050136 of *FTO* with T2DM, and this variant was substantially associated with an increased risk of GDM in a sample of Bangladeshi multigravida women.

## 1. Introduction

Gestational diabetes mellitus (GDM) is a metabolic disorder that affects glucose tolerance during pregnancy, and may resolve after delivery [1]. It is a growing public health concern, with prevalence rate increasing over 30% in recent decades [2]. Expectant mothers with GDM are at risk of various adverse perinatal consequences, such as ketoacidosis, preeclampsia, polyhydramnios, macrosomia, urogenital infection, fetal congenital malformation, dystocia, puerperal infection, neonatal hypoglycemia, respiratory distress syndrome, neonatal low blood calcium disease, and high blood bilirubin [3, 4]. Studies have shown that women with GDM are at an increased risk of developing type 2 diabetes mellitus (T2DM) later in life [5] and their children are more susceptible to diabetes and obesity in adulthood [6]. Hence, it is crucial to identify biomarkers for GDM prevention and screening to improve the lives of affected mothers and infants worldwide.

GDM shares common pathophysiology with T2DM, involving insulin resistance and impaired insulin secretion [7]. Over the past few years, various methods have been employed to elucidate the genetics of GDM and T2DM, such as linkage analysis, candidate gene approach, and genome-wide association studies (GWAS). These studies have revealed that a considerable number of susceptibility genes responsible for T2DM are associated with the risk of developing GDM [8–11]. Positive associations were observed for genes encoding glucokinase [12], calpain-10 [13], sulfonylurea receptor 1 [14], potassium inwardly-rectifying channel, subfamily J, member 11 (*KCNJ11*) [15], β3 adrenergic receptor [16], plasminogen activator inhibitor 1 [17], transcription factor 7-like 2 (*TCF7L2*) [18, 19], and variants of the fat mass associated (*FTO*) gene [8, 20–26].

The *FTO* gene, is located on 16q12.2 (NCBI Gene ID: 79068) [27] and encodes an enzyme, 2-oxoglutarate-dependent nucleic acid demethylase, which is primarily expressed in the hypothalamus [28], and mediates oxidative demethylation of different RNA species involved in fatty acid metabolism, DNA repair, and post-translational modification [29]. Recent studies have shown that *FTO* plays a crucial role in regulating body weight. The enzyme was found to be upregulated in the hypothalamus of food deprived mice and acts as a regulator of fat mass by lipolysis, adipogenesis, and energy homeostasis [30, 31].

Studies have indicated that polymorphisms in *FTO* confer a higher risk of T2DM and GDM across various ethnic groups. In particular the tag-SNP rs8050136, located in intron 1 of *FTO*, is a binding site for both the activator and repressor of the gene [32]. This SNP is in high linkage disequilibrium (LD) with five other SNPs (r^2^ ≥ 0.88) that are associated with obesity and T2DM risk [33]. Studies have shown that this variant is associated with diabetes in a BMI-dependent manner in Caucasians sand [20, 21],Asian populations [34–37] as well as Pima Indians [38]. However, studies on East Asians revealed that rs8050136 is associated with an increased risk of T2DM, independent of BMI [39, 40]. A study on the Sikh population found *FTO* variants to be a risk factor for T2DM with a weak association with BMI [41]. In a small European population the association between T2D and the risk allele (A) was weakened but not abolished after adjusting for BMI [42]. Studies have found that the SNP was linked to an increased risk of postpartum T2DM development in mothers with GDM in Europe [43]. *FTO* SNP rs8050136 is also significantly associated with HbA1C and fibrinogen, which are prominent markers of glucose homeostasis [44]. Subjects with the risk allele (A) of the SNP were observed to have high insulin resistance and increased insulin secretion [45]. However, several studies have reported no association between *FTO* rs8050136 and T2D or GDM. Studies in the Euro-Brazilians [46], a European population [44], a Russian population [47], and multiethnic populations from North America [48–50] have shown no association with T2D. Similar results were obtained from studies on Asian populations with different ethnicities, including, East Asian, South Asian, and Middle Eastern [51–53]. Studies on Mexicans [54], Koreans [55] and Euro-Brazilians [56] concluded that rs8050136 is not significantly linked to GDM etiology. In contrast, a study on the South African Black population has found that the polymorphic variant has a protective effect against T2D [57].

Although an increasing number of studies on the association between *FTO* SNP rs8050136 and T2DM, as well as GDM risk have been reported, the outcomes of these studies were incongruous owing to differences in genetic and anthropometric aspects between Asian and non-Asian populations. For instance, previous studies have revealed that Asian women with GDM typically have lower BMI than their non-Asian counterparts [58, 59]. As *FTO* variants are speculated to be associated with T2D in an adiposity-dependent manner, further investigations on Asians are warranted to elucidate how the genetics of *FTO* contributes to GDM. Despite a growing body of literature on this topic, few studies have examined the association between the variant rs8050136 and GDM; and to the best of our knowledge, no study on the Bangladeshi population has been conducted. To address this gap, we conducted a case-control study of Bangladeshi GDM patients to explore the potential association between this T2D linked SNP and GDM in this population. Additionally, we performed a comprehensive review and meta-analysis, to summarize all relevant evidence and gain a better understanding of the relationship between the aforementioned *FTO* variant and T2D risk. Our study aims to contribute to the existing body of knowledge on this topic and to encourage future research.

## 2. Materials and methods

### 2.1 Meta-analysis

The study conformed to the Preferred Reporting Items for Systematic Review and Meta-Analysis Protocol (PRISMA) guidelines [60]. A checklist is provided in S2 File.

#### 2.1.1 Data retrieval strategy

**Fig 1:**
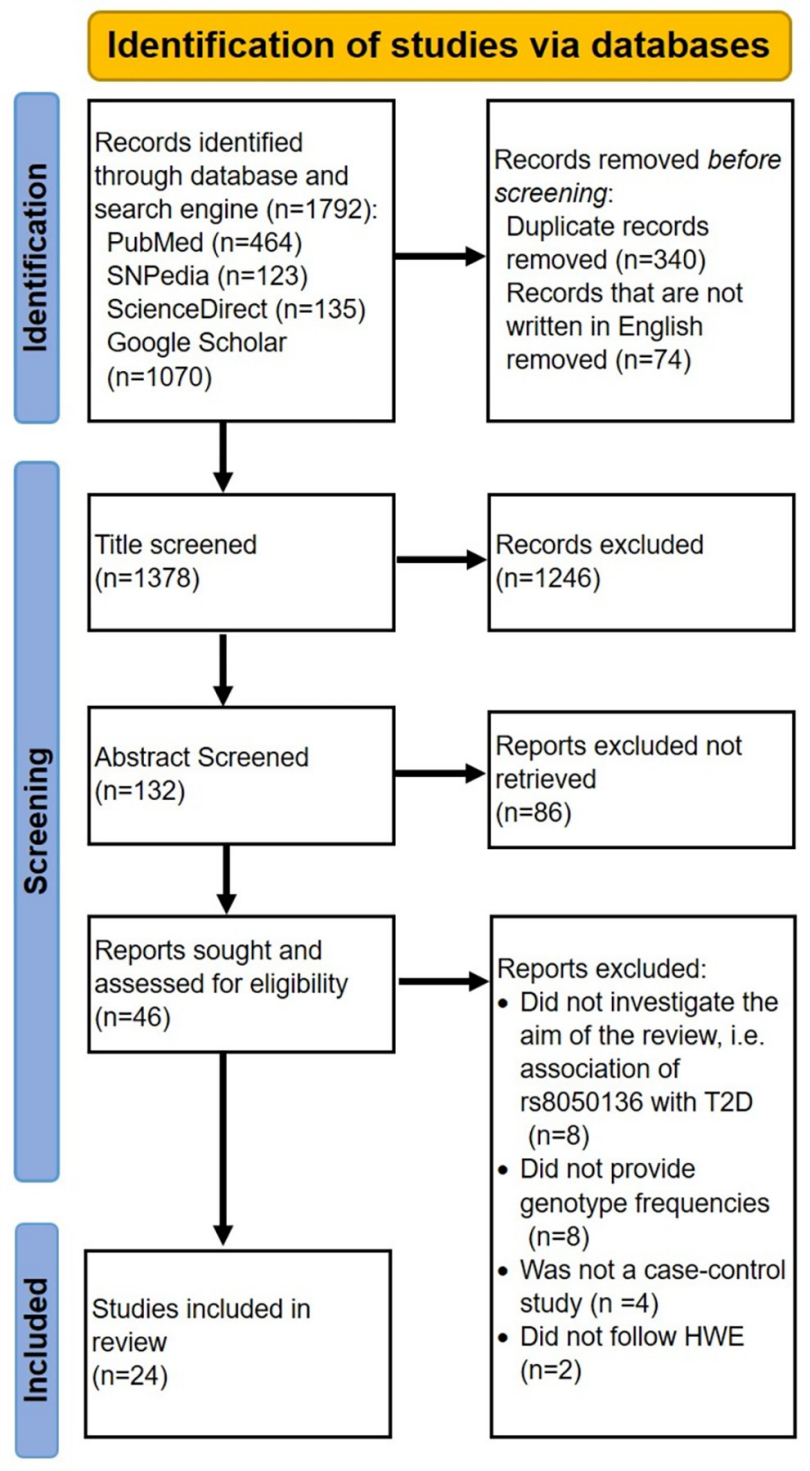
The flow diagram illustrating the literature search strategy includes the number of records identified, screened, included as well as excluded with respective reasons, conforming to PRISMA guidelines.

A thorough literature mining was carried out using electronic bibliographic databases, including PubMed, ScienceDirect, and the search engine Google Scholar, as well as studies mentioned in SNPedia (Fig 1). The search was conducted using the following keywords: “*FTO* rs8050136” OR “rs8050136” AND “Type 2 Diabetes” OR “T2DM”. The final search was conducted on June 2^nd^ 2022. The language of the articles was limited to the English language. The studies included in the meta-analysis studies were manually examined to avoid omitting important papers.

#### 2.1.2 Inclusion and exclusion criteria

Selected publications had to meet the following inclusion criteria: (1) studies investigating the association of *FTO* rs8050136 polymorphism with T2DM; (2) case-control studies, case-cohort and cross-sectional studies were considered; (3) studies that provided odds ratio (OR) with 95% confidence intervals (CI), *P*-value, and genotype frequencies; (4) publications from January 2007 to June 2022. Articles with the following criteria were excluded: (1) Articles written in any language other than English; (2) any reviews, letters, or meta-analyses; (3) articles unrelated to T2DM or *FTO* rs8050136; and (4) studies where homozygous and heterozygous genotypes could not be ascertained.

#### 2.1.3 Data extraction

Data were extracted twice: once manually and once using the Harzing publish or perish (PoP) software [61]. The publications which met the inclusion criteria described above were thoroughly scrutinized. Following are the data types which were extracted: DOI, type of study undertaken, title of the paper, date, genotyping method, number of cases and controls involved in the study, their genotype frequencies, nationality and ethnicity of the subjects, and information on age and BMI.

#### 2.1.4 Quality assessment and HWE conformance of the studies

The quality of each study was evaluated via the Newcastle-Ottawa Scale (NOS) [62]. Each study went through scrutiny under three criteria, 1) Selection. This was based on the selection of case and control of the studies, whether the cases were representative of the general population and how they were ascertained as cases, and whether the controls belong to the same community as the cases. 2) Comparability. This includes any adjustments for confounding variables, such as age, sex, and BMI, while conducting the statistical analyses. 3) Exposure. We analyzed whether the subject underwent the same assessment to be assigned as cases or control and whether there was any information on non-response. Studies with a score from 7–9, have high quality, 4–6, have a risk of bias, and 0–3 have a very high risk of bias. Almost all the studies in our analyses had scores between 6 and 8, and only one had a score of 4. The selected studies were also checked for conformance to the Hardy-Weinberg Equilibrium (HWE) by chi-square test. The *P* values obtained were corrected for multiple testing using FDR (False Discovery Rate) method. If the *P*-value remained significant, control genotypes may not have been in HWE and the study was excluded from the analyses.

#### 2.1.5 Statistical analyses

To evaluate the relationship of *FTO* rs8050136 polymorphism with T2DM risk, a meta-analysis was conducted using a series of genetic models, including the dominant model (AA+AC vs. CC), recessive model (AA vs. AC+CC), overdominant model (AC vs. AA+CC), allele model (A vs. C), homozygous model (AA vs. CC), and heterozygous model (AA vs. AC and AC vs. CC). In addition to this, ethnicity and country-based subgroup analyses were carried out. The strength of correlation between *FTO* rs8050136 and T2DM was measured by odds ratios and the corresponding 95% confidence intervals (CIs). A z-test was performed to estimate the significance of the pooled ORs [63]. Between-study heterogeneity was evaluated by the Cochran’s χ2-test-based Q test and I^2^ statistics [64]. *P* value of Q-test < 0.10 and I^2^ > 50% indicated evidence of heterogeneity, and then a random-effects model [63] was used to count the summary risk estimate; otherwise, the fixed-effect model [65] was performed. Egger’s test for asymmetry along with Begg’s funnel plot was used to estimate the potential publication bias [66]. For meta-analysis, statistical analyses were performed using the web tool MetaGenyo, where *P* values were 2-sided with a statistical significance level of 0.05, except for tests of heterogeneity where 0.10 was selected as the significance level [67]. The sensitivity of analysis was executed by omitting each study individually and conducting all calculations using the remaining studies to appraise the robustness of the pooled results.

### 2.2 Case-control study

#### 2.2.1 Study subjects

A case-control study was conducted, involving 502 unrelated pregnant women (218 with GDM and 284 controls) in any trimester who attended an antenatal clinic for their regular checkups and treatment at the Department of Gynecology and Obstetrics, Bangabandhu Sheikh Mujib Medical University (BSMMU). Participants with known diabetes mellitus, acute critical illness, steroid medication for any reason, and established organ dysfunction, such as known cases of thyroid dysfunction, chronic renal disease, chronic liver disease, or heart failure, were excluded. Informed consent was obtained from each participant, and the study protocol (NIBREC 2016–04) was approved by the research ethics committee (REC) of the National Institute of Biotechnology (NIB).

#### 2.2.2 Body mass index

Standardized protocols were used to acquire anthropometric measurements of weight and height. In both GDM patients and normoglycemic pregnant controls, the body mass index (BMI) was calculated by dividing the weight in kilograms by the square of the height in meters.

#### 2.2.3 Oral Glucose Tolerance Test (OGTT)

After an overnight fast of 8–10 hours, participants underwent a 75 g OGTT on the designated day, and their glycemic status was determined using the WHO 2013 criterion for GDM [fasting plasma glucose (FPG) 5.1–6.9 mmol/l and/or 1-h plasma glucose (OPG) 10.0 mmol/L and/or 2-h plasma glucose (TPG) 8.5–11.0 mmol/L]. Commercial kits were used to assay plasma glucose by the glucose oxidase method in an automated analyzer (Dade Behring, Germany). If the glycemic status was found to be normal when tested before the 24th week of gestation, the mother was advised to repeat the OGTT during the 24th to 28th week of gestation, and the glycemic status was reconsidered.

#### 2.2.4 DNA extraction

Peripheral blood samples of patients and controls were collected in BD Vacutainer^®^ K2 EDTA (BD Franklin Lakes NJ USA) tubes. Genomic DNA was extracted from this blood sample using the PureLink^®^ Genomic DNA extraction kit (Invitrogen) according to the manufacturer’s instructions. The concentration and purity of the extracted DNAs were tested with NanoDrop 2000 UV Vis Spectrophotometer. DNA samples with an OD_260_/OD_280_ ratio between 1.8–1.85 and concentrations more than 80 ng/mL were used for genotyping.

#### 2.2.5 Tetra-primer Amplification Refractory Mutation System Polymerase Chain Reaction (T-ARMS-PCR)

**Fig 2:**
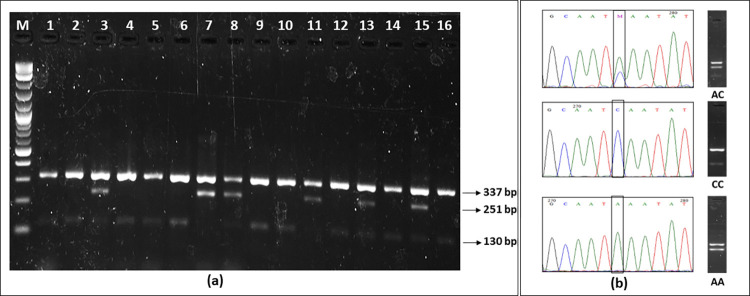
(a) Lane1 (M): 1Kb+ DNA Ladder. Lane 1, 2, 4, 5, 6, 9, 10, 12, 14, 16 homozygous for wild type (CC). Lanes 3, 8, 13, and 15 are heterozygous (AC). Lanes 7 and 11 are homozygous for the mutant allele (AA). (b) Validation of the T-ARMS results by DNA sequencing.

The T-ARMS-PCR method [68] was employed to genotype the rs8050136 polymorphism of the *FTO* gene. The primers were designed using the Primer1 program [69]. The target region was amplified in a single reaction using the following primers: outer Forward 5′-CTTAAGAGTCCATACCAACCAAGGT-3′; outer Reverse 5′ATAATTGGCTCTCGACATT TACACA-3′; inner Forward 5′-AGTTGCCCACTGTGGCAGTC-3′ and inner Reverse 5′-GCAAAAACCACAGGCTCAGATACTT-3′. To increase the specificity of the reaction, the inner primers were designed to have a mismatch at the −2 position from the 3′ terminal. The PCR cycles consisted of an initial denaturation step of 5 min at 95°C, followed by 30 cycles of 50s at 95°C, 45 s at 62°C, and 45s at 72°C. The final extension was for 7 min at 72°C. Two nonallele specific outer primers amplify 337 bp region that comprises the SNP, while two allele specific inner primers paired with each of the outer primers, and produce the allele specific fragments 251 bp and 130 bp. Subsequently, 7μl of the amplified product was subjected to 2% agarose gel electrophoresis and stained with ethidium bromide. The appearance of two bands of 337 bp and 251 bp indicated the presence of AA genotype, whereas 337 bp and 130 bp products indicated the CC genotype. When the heterozygous genotype AC is present, three bands are visible: 337 bp, 251 bp, and 130 bp [Fig 2(A)].

#### 2.2.6 DNA sequencing

Sanger’s di-deoxy chain-terminating method was used to sequence the amplified *FTO* locus. Chain terminating cycle sequencing for both outer forward and outer reverse primer was performed using a sequencing kit (Big Dye Terminator v3.1, Applied Biosystems, Foster City, Calif. USA). Capillary electrophoresis was performed using the ABI 3500 Genetic Analyzer (Applied Biosystems, Foster City, Calif. USA). Sequences were analyzed to determine the genotype using sequencing analysis software, version 5.2 (Applied Biosystems, Foster City, Calif. USA). T-ARMS-PCR genotyping results were validated by sequencing blindly selected samples [Fig 2(B)]. No differences were found thus the genotyping error rate was 0%. Some of the sequences submitted to NCBI GenBank under the accession numbers OP313800, OP589308, OP615665, OP615666, OP615667, OP615668, OP615669, OP615670, OP629686, OP629687, OP629688, OP629689, OP629690, OP629691, OP629692, OP629693, OP629694, OP629695, OP629696, OP629697, OP629698.

#### 2.2.7 Statistical analyses

The normality of the random variables was tested visually by inspecting histograms in R statistical software version 4.0.3 for case control data analysis. Student’s t-tests were used to compare the ages, BMI, systolic blood pressure, diastolic blood pressure, and plasma glucose levels (FPG, OPG, and TPG) between the GDM and control groups, while the chi-square test was used to examine the family history of diabetes and gravidity between these groups. Numerical variables were expressed as mean ± standard deviation of the mean (mean ± SD). Categorical variables, on the other hand, were given as numbers (n) and percentages (%). Hardy-Weinberg equilibrium (HWE) was assessed in cases and controls separately using Pearson’s chi-squared (χ^2^) test with a *P*>0.05 criterion. Multivariate logistic regression was used to identify GDM risk variables. The general relationship of genotypes with GDM was examined with multivariate logistic regression analysis under codominant, dominant, recessive, overdominant, and log-additive models and adjusted for family history of diabetes and gravidity using SNPStats [70]. The best-fitting model with numerous variables was determined by adding prospective confounding factors progressively using Akaike’s Information Criterion (AIC) and Bayesian Information Criterion (BIC) values, which are a goodness of fit criteria.

## 3. Result

### 3.1 Meta-analysis

#### 3.1.1 Non-biased high-quality data from several ethnic populations from different countries retrieved for meta-analysis

A total of 1792 articles were initially identified from electronic databases (Fig 1). Following the removal of duplicates (n = 340) and articles not written in English (n = 74), 1378 studies were eligible for title screening. A total of 1246 papers were excluded after screening the titles, leaving 132 articles for abstract screening. Eventually, 46 articles were selected to screen for eligibility criteria. After omitting 8 papers for reporting the association with GDM or obesity or SNPs other than rs8050136, another 8 for not providing enough information to ascertain homozygotes and heterozygotes genotype frequencies, and 4 for being GWA studies or gene-gene interaction studies, a total of 26 articles were chosen for analysis. Midst these, two studies (Song et al., 2008 [49] and Nikitin et al., 2017 [47]) deviating from the Hardy-Weinberg equilibrium (HWE) were rejected [71]. Finally, 24 papers containing 25 studies were selected and amongst which no paper was published repeatedly, there were no reviews or meta-analyses, and all were involved with *FTO* gene rs8050136 C/A polymorphism and T2DM (Fig 1). Each publication generally included one study, except one, the manuscript published by Bressler, Jan, et al. included two individual studies [72]. The present meta-analysis involved populations from fifteen countries, namely, Bosnia, Brazil, China, Hong Kong, India, Indonesia, Japan, Kazakhstan, Korea, Lebanon, Netherlands, Palestine, Russia, Sweden, USA, with a diverse range of ethnicities.

**Fig 3:**
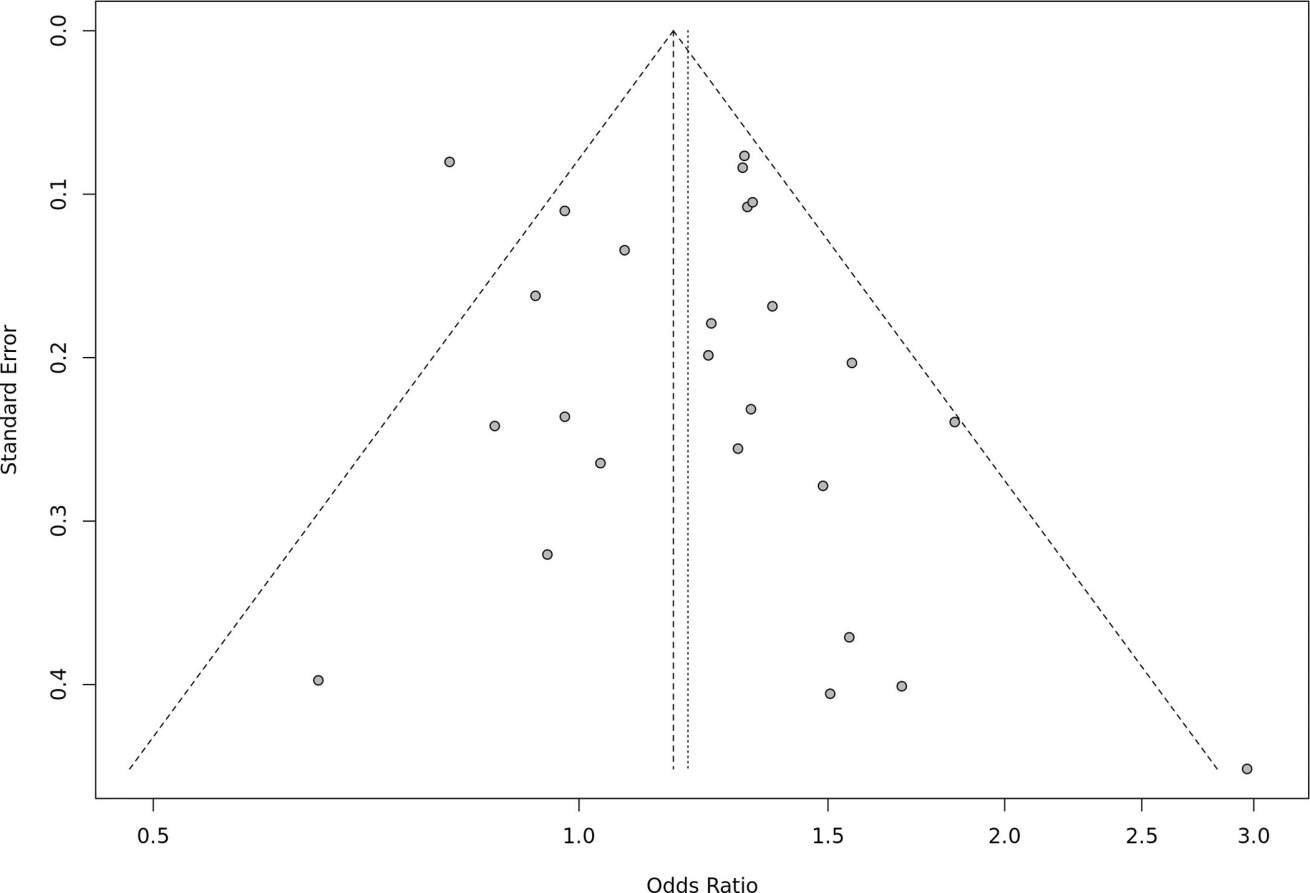
Funnel plot showing the symmetrical distribution of studies indicating an absence of publication bias.

The general characteristics of the studies included in the meta-analysis and the distributions of genotypes in case and control groups are shown in Table 1. The assessment of publication bias revealed a symmetrical shape of Begg’s funnel plot in all models (Fig 3) and *P* values for Egger’s test were greater than 0.05 (S1 Table), signifying that publication bias did not exist. The NOS for assessing the quality of included studies is shown in S2 Table, and the scores ranged from 4 to 8. Only one case–control study was awarded a score of 4; the rest had a score of 6 or above. 11 studies were awarded a score of 8, while seven studies received a 7 NOS score (S2 Table).

**Table 1:** The baseline characteristics of all qualified studies in this meta-analysis.

Study	Year	Country	Ethnicity	AA_Cases	AC_Cases	CC_Cases	AA_Controls	AC_Controls	CC_Controls
Horikoshi,et al.	2007	Japan	Mongolian	37	334	486	38	269	554
Zeggini et al.	2007	UK	European	378	987	550	464	1407	1063
Scott et al.	2007	Finland	European	342	1147	912	398	1104	837
Lee et al.	2008	Korea	Korean	14	200	672	12	116	373
Ng et al.	2008	Hong Kong	Asian	52	760	2223	51	809	2803
Hoek et al.	2008	*Netherlands*	Caucasians	121	302	263	731	2412	2078
Omori et al.	2008	Japan	Asian	84	505	1027	36	339	685
Rong et al.	2009	USA	Pima Indian	44	357	1071	30	435	1360
Han et al.	2010	China	Han Chinese	17	229	761	10	195	790
Wen, et al.	2010	China	Mongolian	19	275	871	12	245	879
Bressler, et al.	2010	USA	African American	126	306	225	533	1334	861
Bressler, et al._1	2010	USA	Caucasian	200	473	311	1614	4714	3545
Chauhan et al.	2011	India	Indo-European	132	455	432	122	437	446
Ramya, et al.	2011	India	South Asian	14	210	627	11	193	797
Ekelund et al.	2012	Sweden	75% Europeans and 25% non-Europeans	25	62	39	77	223	180
Iwata et al.	2012	Japan	Asian	32	233	459	23	234	506
Qian et al.	2013	China	Han Chinese	41	652	2205	35	602	2625
Almawi, et al.	2013	Lebanon	Middle Eastern	253	462	280	220	526	330
Xiao et al.	2015	China	Uyghur	80	332	386	60	279	389
Welter et al.	2016	Brazil	Euro Brazilian	35	84	82	34	89	78
Xiao, et al._1	2016	China	Uyghur	89	372	418	68	357	470
Sikhayeva, et al.	2017	Kazakhstan	Middle Eastern	26	174	181	63	299	451
Sharif et al.	2018	Palestine	Palestinian	26	39	35	27	51	22
Adhiyanto et al.	2019	Indonesia	Indonesian	20	16	15	10	19	27
Bego et al.	2019	Bosnia	Bosnian	130	264	133	84	150	89

#### 3.1.2 *FTO* rs8050136 polymorphism significantly increase T2DM risk in Asians

**Fig 4:**
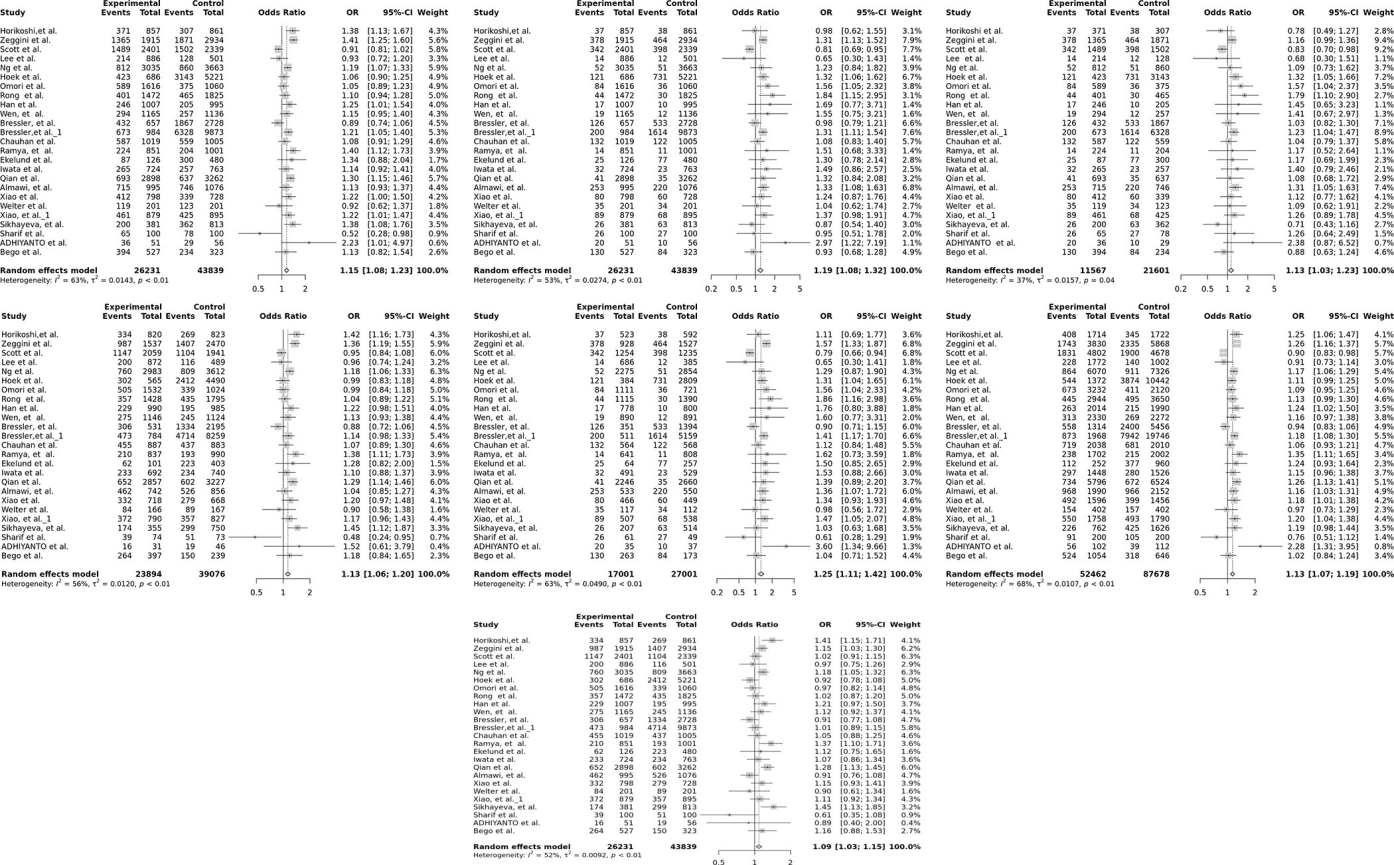
The association of *FTO* rs8050136 polymorphism with T2DM in the overall population under the (a) dominant, (b) recessive, (c) AA vs. AC, (d) AC vs. CC, (e) allele contrast and (f) overdominant models. Pooled OR and 95% confidence intervals (CIs) are shown. Pooled risk estimates were calculated by the random-effects meta-analysis. The squares indicate the odds in a particular study and the horizontal lines represent the corresponding 95% CIs. The diamond indicated the pooled odds ratio.

**Fig 5:**
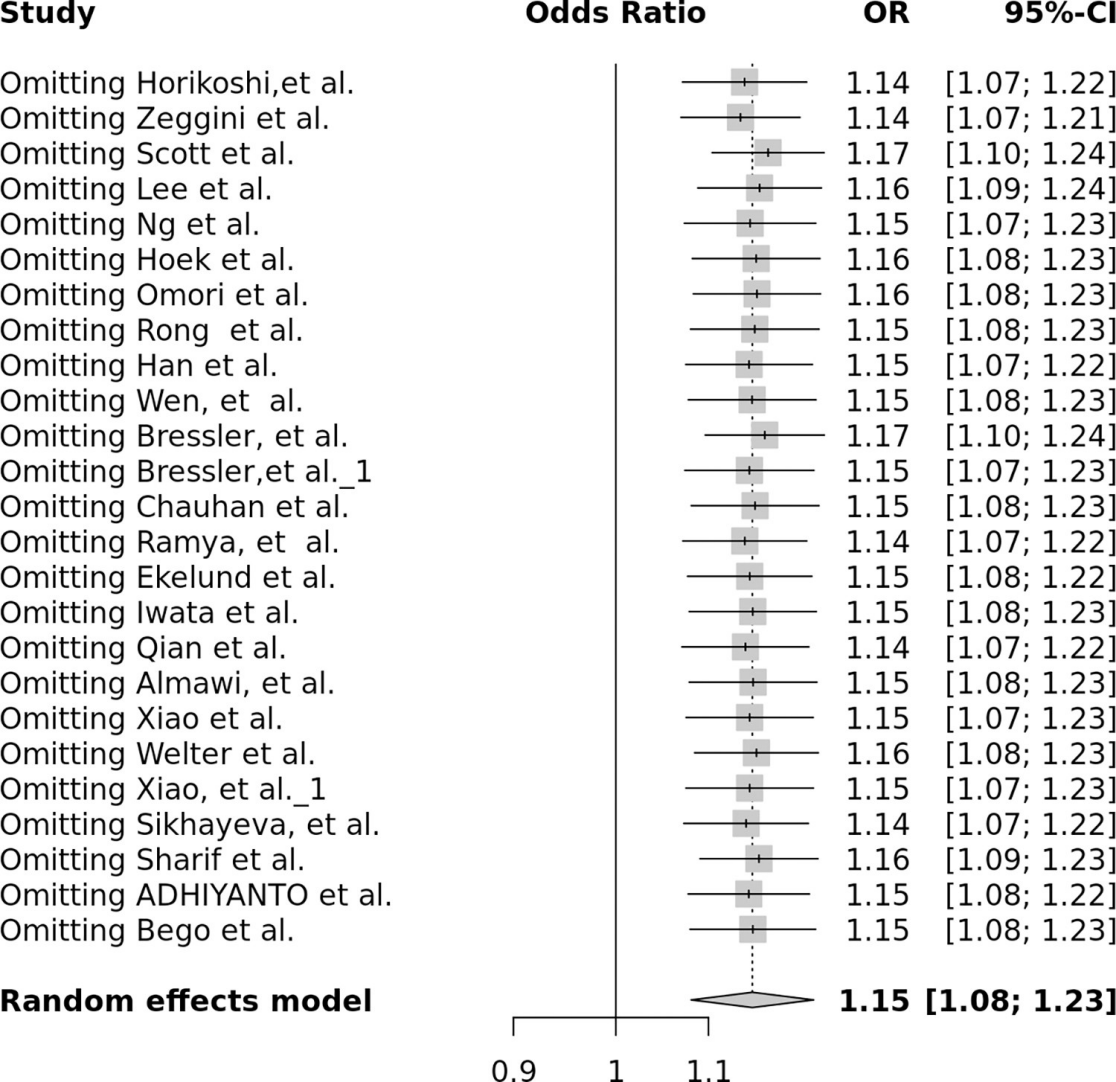
Sensitivity analysis of the association between *FTO* rs8050136 polymorphism and T2DM under the dominant model.

In this study, a total of 70070 samples consisting of 26231 cases and 43839 controls from 24 different publications and 25 different studies were analyzed [20, 21, 34–40, 43–46, 51, 72–81]. The heterogeneity between studies was high, with I^2^ ranging from 52% to 68% for all comparisons except for the AA vs. AC model, where it was 37%, thus the random effect model was employed to calculate the ORs and 95% CIs for all genetic models (S3 Table). Pooled analyses indicated statistically significant associations between the *FTO* rs8050136 polymorphism and increased risk of T2DM (*P*-value <0.05) under all genetic models (the dominant model (Fig 4A), AA+AC vs. CC: OR 1.2; the recessive model (Fig 4B), AA vs. AC+CC: OR 1.2; the AA vs. AC model (Fig 4C): OR 1.13; the AC vs. CC model (4d): OR 1.13; the AA vs. CC model (Fig 4E): OR 1.3; allele contrast, A vs. C (Fig 4F): OR 1.13 and the overdominant model (Fig 4G), OR 1.09 (Fig 4 and S4 Table). Sensitivity analysis was performed by removing each article successively, but the result remained unchanged (Fig 5), indicating the credibility and stability of our findings.

Subgroup analyses were performed to investigate the potential impact of ethnicity and country of origin on the association between *FTO* rs8050136 polymorphism and T2DM risk. The studies included were stratified into Asian and non-Asians groups (S5 Table) and further categorized based on the country where the research was conducted (S6 Table). The results from the sub-group analyses revealed that the *FTO* rs8050136 polymorphism significantly increased T2DM risk in Asians under all genetic models (S5 Table). In the country-based subgroup analyses, only four countries have more than one study which are India (2 studies), Japan (3 studies), USA (3 studies), and China (5 studies). In the Chinese subgroup, the SNP being studied significantly increased T2DM susceptibility under all genetic models except for AA vs. AC. Similarly, in the Japanese subgroup, T2DM risk was found significantly associated with this polymorphism under four genetic models (Allele contrast, Dominant model, Recessive model, and AC vs. CC). In the USA subgroup, the SNP is significantly associated with the risk of T2DM, but only under AA vs. AC model (S6 Table). No evidence of association was observed between *FTO* rs8050136 polymorphism and T2DM risk in the remaining subgroups i.e., non-Asians and Indian subgroups.

### 3.2 Case-control analysis

#### 3.2.1 GDM is significantly higher in Multigravida women

This study enrolled 502 pregnant women classified into 218 with GDM and 284 normoglycemic control subjects. The age of the participants ranged from 18 to 44 (years). The GDM group had significantly(*P*<0.05) higher age (27.57±4.60 vs. 25.46±4.78), BMI (26.64±4.03 vs. 25.19±3.84), diastolic blood pressure (DBP: 70.65±9.16 vs. 68.83±8.99), plasma glucose levels (FPG: 5.15±0.69 vs. 4.33±0.47, OPG: 9.88±1.58 vs. 7.50±1.22, and TPG: 8.26±1.48 vs. 6.43±1.05); a higher percentage of positive family history of diabetes (FH: 46.58% vs. 30.77%, *P* = 0.0003), multigravida (62.84% vs. 53.52%, *P =* 0.032) and lower percentage of primigravida (37.16% vs. 46.48%, *P =* 0.032) compared to the control group. However, there was no significant difference between the two groups in terms of systolic blood pressure (SBP: 109.25±11.79 vs. 108.75±11.96, *P* = 0.5744), positive bad obstetric history (BOH: 27.06% vs. 26.06%, *P* = 0.787) and occupation (Housewife: 74.31% vs. 80.28%, Service holder: 25.69% vs. 19.72%, *P* = 0.112). SBP, BOH, and occupation were indecipherable between control and GDM groups whereas significant differences were observed in age, BMI, DBP, FH, and gravidity. Age (OR = 1.1, 95% CI = 1.06–1.14, *P*<0.0001), BMI (OR = 1.09, 95% CI = 1.05–1.15, *P* = 0.00013) and DBP (OR = 1.02, 95% CI = 1.003–1.044, *P* = 0.02) had odds very close to 1, indicating that they were not strong risk factors for GDM. On the other hand, positive family history of diabetes (OR = 1.78, 95% CI = 1.24–2.57, *P*<0.001) and multigravidity (OR = 1.48, 95% CI = 1.03–2.14, *P* = 0.034) were significantly associated with increased risk of GDM. These variables were adjusted for in subsequent analyses after checking their impact on GDM by multivariate logistic regression.

The study involved 502 pregnant participants, of whom 208 were primigravida and 289 were multigravida. The information on gravidity was missing for five participants. The primigravida group comprised 129 controls and 79 GDM, while the multigravida comprised 152 controls and 137 GDM participants. The anthropometric and demographic data of both groups are provided in S7 Table. Multigravida women had significantly (*P*<0.05) higher age BMI, plasma glucose levels and DBP compared to women of primigravida. There was no significant difference in the positive family history of diabetes between the primigravida and the multigravida group. The percentage of GDM was significantly higher in the multigravida group than in the primigravida group (47% vs. 37%, *P =* 0.03), whereas the percentage of normoglycemic women was higher in the primigravida group than in the multigravida group (62% vs. 52%, *P =* 0.03). In both groups, participants with GDM had significantly higher age, BMI, plasma glucose levels, and positive family history of diabetes than control participants (S7 Table).

#### 3.2.2 Genotype frequency and association of *FTO* gene variant with GDM

Genotyping for the *FTO* gene polymorphism showed that the genotype distribution of this SNP differed between those with and without GDM (Table 2). The frequencies of risk genotypes (AC and AA) of this SNP were higher in the GDM group, suggesting the risk-providing nature of this polymorphism. Genotype distributions of cases (GDM) and controls were consistent with Hardy-Weinberg equilibrium (HWE) (Table 2). The minor allele (A) frequency was higher in the GDM group.

**Table 2:** Genotype and allele frequency of *FTO* gene variant rs8050136 in the study participants.

SNP	Genotype/Allele	Control (%)	GDM (%)
**rs8050136**	CC	143 (50.35%)	101 (46.33%)
	AC	126 (44.37%)	98 (44.95%)
	AA	15 (5.28%)	19 (8.72%)
	C	412(72.54%)	300(68.81%)
	A	156(27.46%)	136(31.19%)
**HWE**	Chi-Square (χ^2^)	1.29	0.22
	*P* value	0.26	0.64

If *P* < 0.05—not consistent with Hardy Weinberg Equilibrium (HWE).

**Fig 6:**
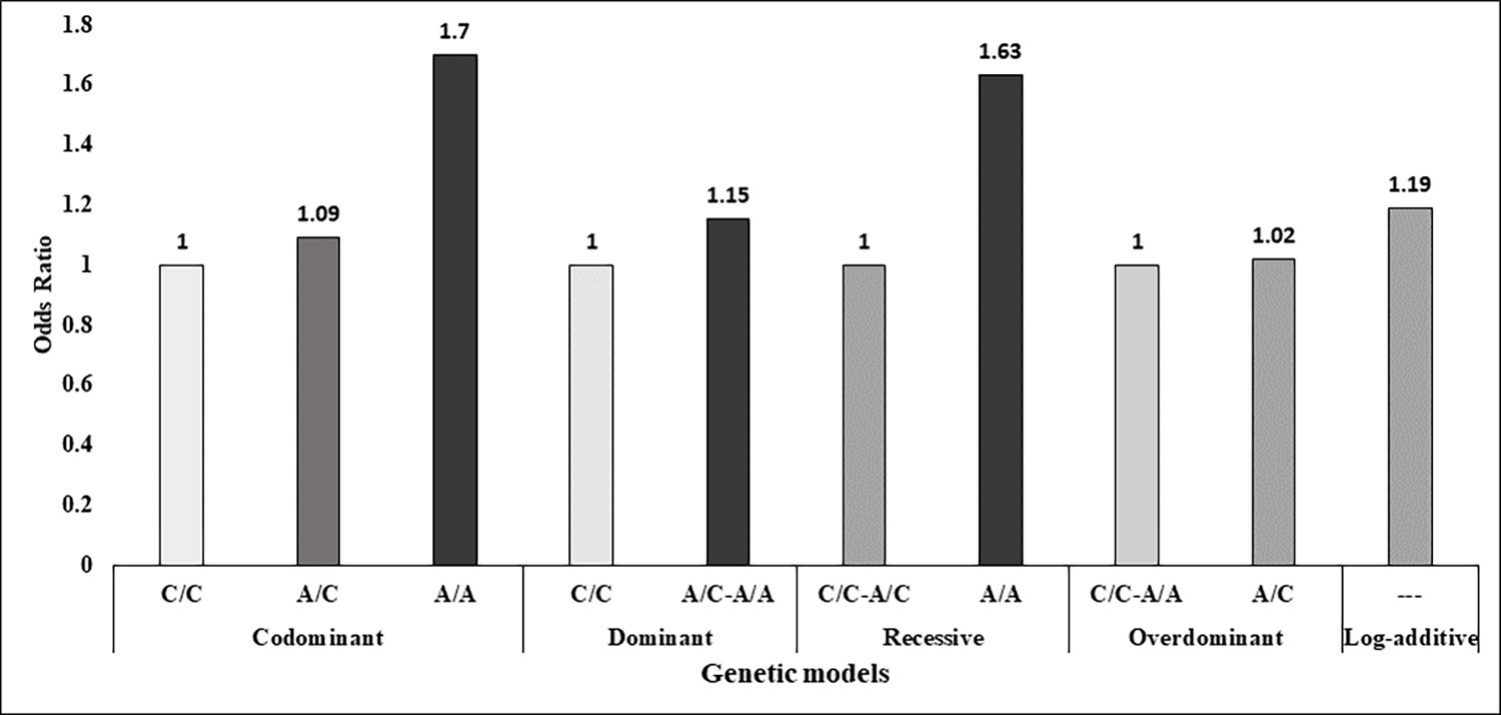
Association of rs8050136 (CC/AC/AA) with GDM under different genetic models adjusted for family history of diabetes and gravidity.

The association of this genetic variant with GDM was tested under codominant, dominant, recessive, overdominant, and log additive models (S8 Table). Analyses revealed a greater than 1.6 folds increase in odds of developing GDM under codominant [C/C vs. A/A (OR = 1.70, 95% CI = 0.81 to 3.55), *P* = 0.37] and recessive (OR = 1.63, 95% CI = 0.80 to 3.33, *P* = 0.18) models after adjusting for family history of diabetes and gravidity. In the rest of the models, the odds remained between 1.1 and 1.2, with *P* values greater than 0.05 (Fig 6). None of the five genetic models showed a significant difference in the likelihood of developing GDM before this adjustment (S8 Table). The frequency of the A allele of rs8050136 was higher in GDM compared to control, while that of the C allele was higher in control with comparison to GDM (GDM vs. control, A-allele: 31.19% vs. 27.46%; C-allele 68.81% vs. 72.54%) (Table 2). In the presence of the A allele, the odds of having GDM increase by 1.2 folds with a 95% CI of 0.91 to 1.57.

#### 3.2.3 Family history of diabetes and *FTO* rs8050136 increase the risk of GDM

**Fig 7:**
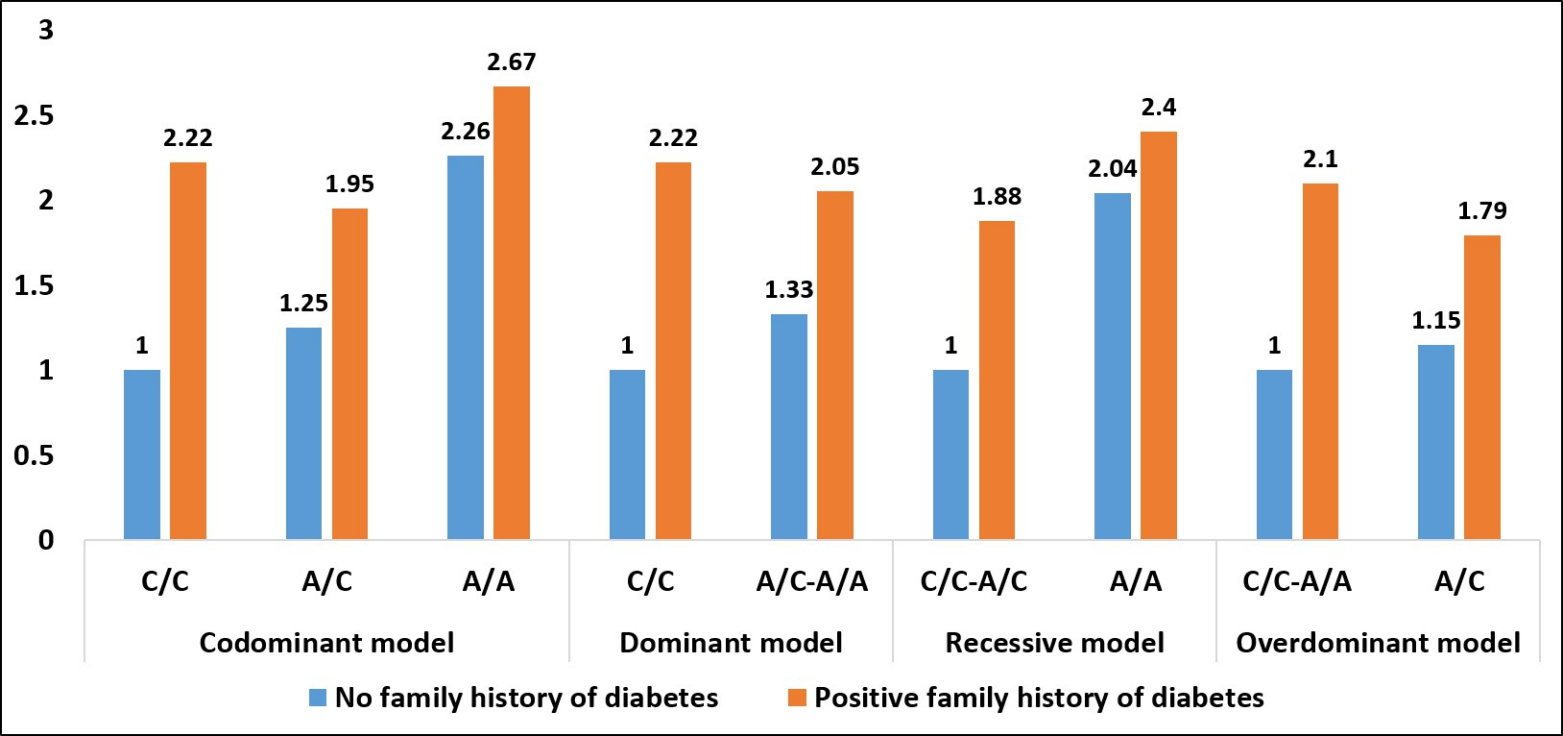
Correlation between GDM risk with cumulation of the FTO gene rs8050136 polymorphism, and family history of diabetes under different genetic models and adjusted for gravidity.

The combined impact of both a family history of diabetes and the rs8050136 variant of the *FTO* gene was found to increase the odds of developing GDM under all tested genetic models, after adjusting for gravidity. Even individuals with a CC genotype exhibited a 2.22 folds increase in odds (95% CI = 1.3–3.8) under codominant and dominant models (Fig 7). In the presence of both a positive family history of diabetes and the AA genotype, the risk of GDM increased from 2.26 to 2.67 under codominant and from 2.04 to 2.40 under recessive models, respectively (S9 Table).

#### 3.2.4 Multigravidity and *FTO* gene rs8050136 variant significantly increase odds of GDM

**Fig 8:**
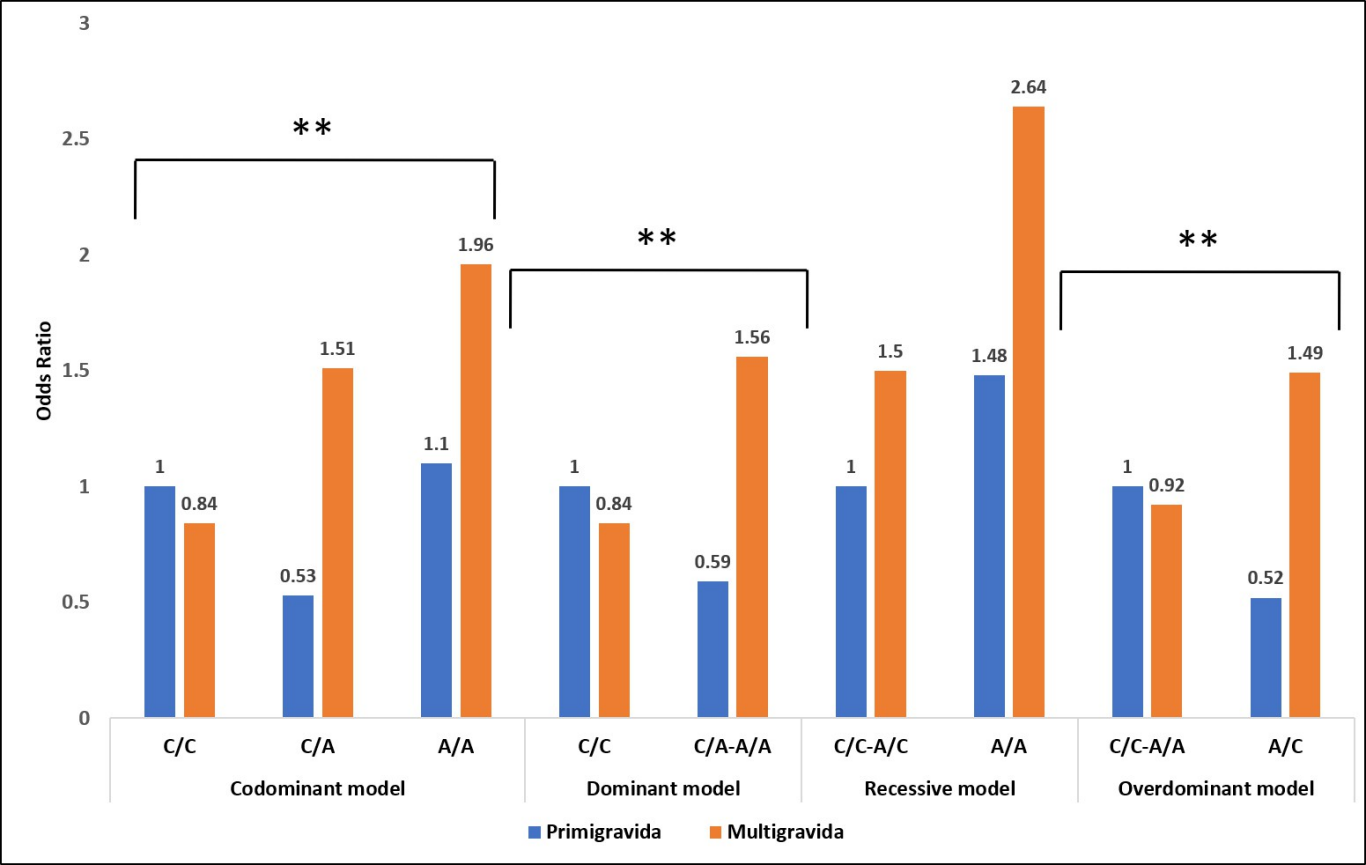
Correlation between GDM risk with cumulation of the *FTO* gene rs8050136 polymorphism, and gravidity. (*, **, *** *P*<0.05, *P*<0.01, *P*<0.001).

Interaction analyses of the *FTO* gene variant rs8050136, gravidity, and GDM revealed significant changes in odd ratios under all genetic models except for the recessive model, when adjusted for family history of diabetes (Fig 8). The AC genotype was revealed to be significantly associated with a protective effect against GDM under Codominant (OR = 0.53, 95% CI = 0.29–0.95, *P* = 0.0068) and overdominant (OR = 0.52, 95% CI = 0.29–0.92, *P* = 0.0025) models in primigravida group. A similar protective role was also observed under the dominant model (OR = 0.59, 95% CI = 0.33–1.03) in the same group and was statistically significant (*P* = 0.0021). In contrast, the CC genotype of reference allele showed significant protection against GDM in multigravida under codominant (OR = 0.84, 95%CI = 0.50–1.42, *P* = 0.0068), dominant (OR = 0.84, 95%CI = 0.50–1.42, *P* = 0.0068), and overdominant (OR = 0.92, 95%CI = 0.57–1.49, *P* = 0.0025) models (S10 Table). The presence of this variant in the multigravida group significantly increased the risk of having GDM increased by 1.6 to 2.3 folds under these three models. In multigravida women, the chances of developing GDM increased significantly (*P* = 0.0068) by 1.8 folds (OR = 1.51) in the presence of AC and 2.3 folds (OR = 1.96) in the presence of AA genotypes. The AC genotype significantly increased the odds of GDM in multigravida women by 2.8 and 2.9 folds under codominant and overdominant models, respectively, compared to primigravida women. On the other hand, the AA genotype increased the odds by 1.8 folds under codominant and recessive models (S10 Table). Analysis by recessive model also showed the highest odds (OR = 2.64) though not statistically significant.

#### 3.2.5 Association of rs8050136 with GDM under different genetic models in primigravida and multigravida group

The association of rs8050136 with GDM in both primigravida and multigravida groups has been evaluated using all five genetic models, with a family history of diabetes taken into account. In the Primigravida group, all genetic models, except the recessive model demonstrated protection against GDM by lowering the likelihood of developing the disease. Only the over-dominant model (OR = 0.51, 95% CI = 0.28–0.91, *P* = 0.022) showed a statistically significant reduction both before and after adjustment (S11 Table). In contrast, this variant significantly increased the odds of GDM by 1.6 to 2.4 folds under all tested models, except the recessive model, both before and after adjustment for confounders (S12 Table) in the multigravida group.

#### 3.2.6 Cumulative effect of *FTO* gene variant rs8050136 and family history of T2DM on GDM under different genetic models in primigravida and multigravida women

To detect the cumulative effect of *FTO* gene variant rs8050136 and family history of T2DM on GDM in primigravida and multigravida women of this study a cross-classification interaction has been carried out under codominant, dominant, recessive, and over-dominant models. Our results revealed in primigravida women with positive family history of diabetes, the reference allele of this variant increased the odds of developing GDM by 2.3 to 2.5 times under dominant (OR = 2.50, 95% CI = 1.09–5.72), recessive (OR = 2.26, 95% CI = 1.24–4.12) and over dominant (OR = 2.28, 95% CI = 1.06–4.91) models (S13 Table). Conversely, in multigravida women with a positive family history of diabetes, both the reference and risk alleles increased the odds of developing GDM by 1.7 to 3.4 times under all tested models (S14 Table). None of these changes in odds were statistically significant.

## 4. Discussion

Meta-analyses are conducted to evaluate the reliability of the available evidence, for example, the nature of association in case-control studies [82, 83]. The objective is to determine the existence of any effect, positive or negative, and to obtain a single summary estimate of the effect. In this study, we reviewed 25 eligible articles to assess the effects of *FTO* SNP rs8050136 on susceptibility to T2DM. The meta-analysis of the selected dataset confirmed that the minor A allele of this *FTO* variant may increase the risk of developing T2DM in five genetic models.

The mean of the distribution of true effects was estimated using the random-effects model. Even though the number of cases present in the studies ranged from 51 to 3035 and the number of controls subject varied from 56 to 9873, studies with larger sample sizes provide better estimates than smaller ones. Each study measured a distinct effect size, and each of these effect sizes served as a sample from the population whose mean we have attempted to estimate. As a result, the weights assigned under random effects were more balanced compared to the fixed effects model. Small studies are less likely to be ignored, whereas extensive studies are less inclined to dominate the analysis [84]. Cochran’s χ^2^-test-based Q test and I^2^ were conducted to assess the heterogeneity of the studies [64], for which I^2^ > 50.0% indicates prominent heterogeneity between the studies, thus making the random effect model [63] more suitable for estimating the pooled OR and 95% CI; otherwise, the fixed effect model [65] is more appropriate. Subgroup analysis revealed that the relationship between *FTO* rs8050136 polymorphism and T2DM risk occurred only among Asians and not among non-Asians, which is consistent with the results of previous meta-analyses [85, 86]. Although country-based subgroup analysis revealed a significant association between target SNP and T2DM according to studies conducted in Chinese and Japanese populations, under all genetic models (except AA vs. AC and AC vs. CC), however no significant association was found in Indian studies [36, 81]. Studies performed on the Caucasian and Black populations in the USA did not show any significant association with the SNP under any genetic models (except AA vs. AC) [72], which aligns with our results obtained from ethnicity-based analysis (S6 Table).

The number of association studies between the SNP rs8050136 and GDM is insubstantial. Moreover, a recent meta-analysis reported by H. He et al., 2018 was limited to only Caucasians due to the unavailability of data from other ethnicities, thus the conclusion could not be applied to all populations [87]. Therefore, more association analyses are needed to elucidate the association between this T2DM-linked variant and GDM, as they share a common pathophysiology. Hence, we have performed this case-control association study in a sample of the Bangladeshi population.

The consistency of the genotype distribution of the target SNP with HWE in the case and control groups indicated that there was no selection bias, population stratification, or genotyping error in the study population [88]. Several studies have investigated the association between the aforementioned SNP and T2DM in different populations worldwide [89]. Nevertheless, the same study on GDM is limited [90]. The *FTO* SNP rs8050136, in particular, is not a prominent genetic regulator in the etiology of GDM in the ethnic groups analyzed, but it is located in an area of considerable linkage disequilibrium of the gene’s intron 1 [91]. Although a recent study discovered a link between *FTO* SNPs and the risk of GDM, other studies have found the opposite; therefore, no clear conclusion has been established [8, 55, 92]. In congruence with that of Cho et al. (OR = 1.12, *P* = 0.30) and Saucedo, Renata et al. (OR = 1.11, *P* = 0.86) we found no significant association (*P* = 0.18) between the *FTO* rs8050136 with GDM but odds (OR = 1.63) of this disease is 1.5 folds higher in the population under study [54, 55]. Not only the odds of GDM but also the minor allele frequency of this variant is much higher (0.291) than that of Korean (0.122) and Mexican (0.138) populations. These findings indicated the risk-providing nature of this SNP. On the contrary, in the Korean population rs8050136 does not increase the incidence of GDM, but provides a protective effect against the disorder by improving insulin secretory ability [55].

Multivariate logistic regression analysis identified gravidity (OR  =  1.5) and family history of diabetes (OR  =  1.84) as confounders for higher risk of GDM. This is consistent with previous studies that have identified family history of diabetes and multigravida as risk factors for GDM [93–95]. To control for the effect of these confounding factors the study population was divided into subgroups based on their levels [96, 97]. Cross-classification interactions using different genetic models (S9 and S10 Tables) were employed to reveal the risk of GDM in each group. Interaction analyses showed substantial increases in the odds of developing GDM in both groups, in association to the target SNP. The reference allele of this variant also demonstrated a notable increase in the risk of this disease in the group with a positive family history of diabetes (S9 Table). However, similar to our previous study, this interaction analysis did not reveal any statistically significant association between the SNP and GDM in the presence of a positive family history of diabetes [97]. The wider CI values and insignificant *P*-values in the different models can be attributed to the small sample size of the subgroups (S9 and S10 Tables).

Multigravida women with the *FTO* rs8050136 variant are more susceptible to developing GDM than primigravida women, although *FTO* rs8050136 did not show any statistically significant association with GDM (Fig 6 and S8 Table). However, in the presence of multigravidity, the risk allele of this SNP significantly increased the odds of GDM in the codominant, dominant, and overdominant models (Fig 8 and S10 Table). Conversely, the heterozygous risk allele of this SNP provided statistically significant protection against GDM under codominant (OR = 0.53), dominant (OR = 0.59), and overdominant (OR = 0.52) models in primigravida women (Fig 8 and S10 Table). Our findings from the cumulative association analysis of the target SNP and gravidity with GDM align with those of previous studies conducted on Brazilian, sub-Saharan African, and Asian populations which found that primigravida status may reduce the risk of GDM [98–100]. In an Asian meta-analysis, it was reported that primigravida status was a protective factor for GDM with an OR of 0.55 and 0.79 (*P* < 0.05), which was similar to our findings in the presence of heterozygous risk allele of the target SNP (S10 Table) [100]. Surprisingly, even in the presence of multigravidity, the reference allele provides significant protection (OR = 0.84) against GDM under the codominant and dominant models, contradicting previous reports of the increased likelihood of having GDM in multigravidity [94, 101].

Our separate analyses of the association between this SNP and GDM in primi and multigravida mothers (S11 and S12 Tables), revealed that the odds of this disease were almost similar to those obtained from interaction analyses. This confirmed the protective effect of the heterozygous genotype of this SNP in primigravida women against GDM, and that this was not due to the primigravida itself [100]. In the multigravida group, the odds of GDM increased significantly with this SNP in all tested models except the recessive model, which also confirmed the risk-providing nature of this SNP in multigravida women.

Multigravida is not only an important predictor of GDM but also affects the prevalence of the disease [102, 103]. In a study of 3,447 pregnant women in Bangladesh, Jesmin et al. observed a higher incidence of GDM with increasing numbers of gestations [104]. Similarly, our study revealed a statistically significant difference (*P* = 0.046) between the incidence of GDM in multigravida (47%) and primigravida (38%) groups. Studies conducted in India and Pakistan also showed a lower incidence of GDM in primigravida [98, 105].

However, the meta-analysis of this study has some limitations that should be considered. Drawbacks, such as insufficient sample sizes of some of the studies, selection bias in the recruitment of patients and controls, and the lack of confounder adjustment, might lead to uncertain outcomes. The number of comparable studies that adjusted for the family history of diabetes was insufficient to perform a meta-analysis. On the other hand, the outcomes of case-control analysis might be specific to pregnant women who were recruited from urban areas such as Dhaka, Narayanganj, and Gazipur that may not be representative of the entire population. This could also explain the nature of the association found in this study between the target SNP, gravidity, and, GDM. Even though several risk factors are addressed in this study, other GDM-related factors such as food intake and physical activity could not be considered, due to a lack of information. As a consequence, the interactions between lifestyle factors and this variant in terms of GDM remain unexplored. As the SNP rs8050136 is an intron variant, the molecular mechanism of how they modulate GDM risk remains largely unknown.

Overall, the existing evidence indicates a positive association between the *FTO* gene variant rs8050136 and T2DM risk, especially in Asian populations. In multigravida women, *FTO* rs8050136 significantly increased the risk of GDM, while no evidence of a direct association was found between this variant and GDM risk in our population.

## 5. Conclusion

The current meta-analysis revealed that *FTO* rs8050136 significantly increases the risk of type 2 diabetes in the Asian population under all genetic models. This association was primarily observed in the East-Asian population but not in the South Asian, which might be attributed to its smaller sample size. Additionally, we investigated the association between *FTO* rs8050136 and the risk of GDM in the Bangladeshi population. This revealed that in the presence of multigravidity, the SNP significantly increased the risk of GDM under codominant, dominant, and overdominant models. Therefore, more epidemiological studies are required to confirm this association to establish *FTO* rs8050136 as a biomarker of GDM forewarning in multigravida women. The elucidation of biological underpinnings underlying the association is of utmost importance.

## Supporting information

S1 Table Publication bias by Egger’s test. (DOCX)

S2 Table The Newcastle-Ottawa Scale (NOS) scores of the selected studies. (DOCX)

S3 Table The heterogeneity of the studies. (DOCX)

S4 Table Pooled association analysis. (DOCX)

S5 Table Subgroup analysis based on ethnicity. (DOCX)

S6 Table Subgroup analysis based on country. (DOCX)

S7 Table General characteristics for primigravida and multigravida women. (DOCX)

S8 Table Association of rs8050136 with GDM under different genetic model. ^a^ adjusted for gravidity and family history of diabetes.
(DOCX)

S9 Table Cross classification interaction table of *FTO* variant rs8050136 and family history of T2DM under different genetic model. ^a^ adjusted for gravidity.
(DOCX)

S10 Table Cross classification interaction table of *FTO* variant rs8050136 and gravidity under different genetic models. ^a^ adjusted for family history of diabetes.
(DOCX)

S11 Table Association of rs8050136 with GDM under different genetic models in primigravida women; N = 208. ^a^ adjusted for family history of diabetes.
(DOCX)

S12 Table Association of rs8050136 with GDM under different genetic models in multigravida women; N = 289. ^a^ adjusted for family history of diabetes.
(DOCX)

S13 Table Cross classification interaction table of *FTO* variant rs8050136 and family history of T2DM under different genetic model in primigravida group. (DOCX)

S14 Table Cross classification interaction table of *FTO* variant rs8050136 and family history of T2DM under different genetic model in multigravida group. (DOCX)

S15 Table Excluded papers that have been sought and assessed for eligibility and reasons for exclusion. (DOCX)

S1 File Meta-analysis on genetic association studies checklist | PLOS ONE. (DOCX)

S2 File PRISMA 2020 checklist. (DOCX)

S1 Fig Uncropped and unadjusted gel image of Fig 2. (PNG)
