# Supplementary material for: Type 2 diabetes linked FTO gene variant rs8050136 is significantly associated with gravidity in gestational diabetes in a sample of Bangladeshi women: Meta-analysis and case-control study
Source: PLoS One. 2023 Nov 30;18(11):e0288318. doi: 10.1371/journal.pone.0288318 (PMC10688623; doi:10.1371/journal.pone.0288318)
Supplement: S1 Table — (DOCX) [file pone.0288318.s001.docx]

**S1 Table. Publication bias by Egger’s test**

| **Genetic models** | ***P*-value** |
| --- | --- |
| Allele contrast  (A vs. C) | 0.6345 |
| Dominant model  (AA+AC vs. CC) | 0.8056 |
| Recessive model  (AA vs. AC+CC) | 0.304 |
| Overdominant model  (AC vs. AA+CC) | 0.5462 |
| AA vs. CC | 0.4762 |
| AA vs. AC | 0.4692 |
| AC vs. CC | 0.5222 |
