# Supplementary material for: Type 2 diabetes linked FTO gene variant rs8050136 is significantly associated with gravidity in gestational diabetes in a sample of Bangladeshi women: Meta-analysis and case-control study
Source: PLoS One. 2023 Nov 30;18(11):e0288318. doi: 10.1371/journal.pone.0288318 (PMC10688623; doi:10.1371/journal.pone.0288318)
Supplement: S2 Table — (DOCX) [file pone.0288318.s002.docx]

**S2 Table: The Newcastle-Ottawa Scale (NOS) scores of the selected studies**

| Study | Year | Selection (Score out of 4) | Comparability (2) | Exposure (3) | NOS SCORE |
| --- | --- | --- | --- | --- | --- |
| Adhiyanto  et al. | 2019 | 2 | 1 | 1 | 4 |
| Hoek et al. | 2008 | 2 | 2 | 2 | 6 |
| Xiao et al. | 2015 | 3 | 1 | 2 | 6 |
| Welter  et al. | 2016 | 3 | 1 | 2 | 6 |
| Sikhayeva, et al. | 2017 | 2 | 2 | 2 | 6 |
| Sharif et al. | 2018 | 2 | 2 | 2 | 6 |
| Bego et al. | 2019 | 2 | 2 | 2 | 6 |
| Wen, et  al. | 2010 | 3 | 2 | 2 | 7 |
| Bressler,et al. | 2010 | 3 | 1 | 3 | 7 |
| Bressler,et al._1 | 2010 | 3 | 1 | 3 | 7 |
| Ramya, et  al. | 2011 | 3 | 2 | 2 | 7 |
| Ekelund et al. | 2012 | 3 | 2 | 2 | 7 |
| Qian et al. | 2013 | 3 | 2 | 2 | 7 |
| Omori et al. | 2008 | 3 | 2 | 2 | 7 |
| Horikoshi,et al. | 2007 | 4 | 1 | 3 | 8 |
| Lee  et al. | 2008 | 4 | 2 | 2 | 8 |
| Ng et al. | 2008 | 4 | 2 | 2 | 8 |
| Rong  et al. | 2009 | 4 | 2 | 2 | 8 |
| Han et al. | 2010 | 4 | 2 | 2 | 8 |
| Chauhan et al. | 2011 | 4 | 2 | 2 | 8 |
| Almawi, et al. | 2013 | 4 | 2 | 2 | 8 |
| Xiao, et al | 2016 | 4 | 2 | 2 | 8 |
| Iwata et al. | 2012 | 4 | 2 | 2 | 8 |
| Zeggini et al. | 2007 | 4 | 2 | 2 | 8 |
| Scott et al. | 2007 | 4 | 2 | 2 | 8 |
