# Supplementary material for: Type 2 diabetes linked FTO gene variant rs8050136 is significantly associated with gravidity in gestational diabetes in a sample of Bangladeshi women: Meta-analysis and case-control study
Source: PLoS One. 2023 Nov 30;18(11):e0288318. doi: 10.1371/journal.pone.0288318 (PMC10688623; doi:10.1371/journal.pone.0288318)
Supplement: S3 Table — (DOCX) [file pone.0288318.s003.docx]

**S3 Table: The heterogeneity of the studies**

| **Genetic models** | **tau^2^** | **H** | **I^2^** | **Q** | ***P*-value** |
| --- | --- | --- | --- | --- | --- |
| Allele contrast  (A vs. C) | 0.01 | 1.76 | 0.68 | 74.54 | 0.00 |
| Dominant model  (AA+AC vs. CC) | 0.03 | 1.46 | 0.53 | 51.23 | 0.00 |
| Recessive model  (AA vs. AC+CC) | 0.01 | 1.63 | 0.63 | 64.07 | 0.00 |
| Overdominant model  (AC vs. AA+CC) | 0.01 | 1.45 | 0.52 | 50.46 | 0.00 |
| AA vs. CC | 0.05 | 1.64 | 0.63 | 64.54 | 0.00 |
| AA vs. AC | 0.02 | 1.26 | 0.37 | 37.94 | 0.04 |
| AC vs. CC | 0.01 | 1.51 | 0.56 | 54.70 | 0.00 |
