# Supplementary material for: Type 2 diabetes linked FTO gene variant rs8050136 is significantly associated with gravidity in gestational diabetes in a sample of Bangladeshi women: Meta-analysis and case-control study
Source: PLoS One. 2023 Nov 30;18(11):e0288318. doi: 10.1371/journal.pone.0288318 (PMC10688623; doi:10.1371/journal.pone.0288318)
Supplement: S4 Table — (DOCX) [file pone.0288318.s004.docx]

**S4 Table: Pooled association analysis**

| **Model** | **Study** | **Number of studies** |  | **Test of association** |  |  | **Test of heterogeneity** |  | **Publication bias** |
| --- | --- | --- | --- | --- | --- | --- | --- | --- | --- |
|  |  |  | **OR** | **95% CI** | ***P*-value** | **Model** | ***P*-value** | **I^2^** | ***P*-value (Egger's test)** |
| **Allele contrast**  **(A vs. C)** | Overall | 25 | 1.13 | 1.0732-1.1916 | 4.08E-06 | Random | 0 | 0.678 | 0.63 |
| **Recessive model**  **(****AA vs. AC+CC)** | Overall | 25 | 1.2 | 1.0804- 1.3201 | 0.00051 | Random | 0.001 | 0.531 | 0.30 |
| **Dominant model**  **(AA+AC vs. CC)** | Overall | 25 | 1.2 | 1.0806-1.2256 | 1.23E-05 | Random | 0 | 0.625 | 0.81 |
| **Overdominant model**  **(AC vs. AA+CC)** | Overall | 25 | 1.09 | 1.0293-1.1493 | 0.00282 | Random | 0.0012 | 0.524 | 0.55 |
| **pairw1**  **(AA vs. CC)** | Overall | 25 | 1.3 | 1.1103- 1.4155 | 0.00026 | Random | 0 | 0.628 | 0.48 |
| **pairw2**  **(****AA vs. AC)** | Overall | 25 | 1.13 | 1.0291-1.2315 | 0.0097 | Random | 0.035 | 0.368 | 0.47 |
| **pairw3**  **(****AC vs. CC)** | Overall | 25 | 1.13 | 1.0615-1.1990 | 0.00010 | Random | 0.000 | 0.561 | 0.52 |
