# Supplementary material for: Type 2 diabetes linked FTO gene variant rs8050136 is significantly associated with gravidity in gestational diabetes in a sample of Bangladeshi women: Meta-analysis and case-control study
Source: PLoS One. 2023 Nov 30;18(11):e0288318. doi: 10.1371/journal.pone.0288318 (PMC10688623; doi:10.1371/journal.pone.0288318)
Supplement: S5 Table — (DOCX) [file pone.0288318.s005.docx]

**S5 Table: Subgroup analysis based on ethnicity**

| **Model** | **Ethnicity** | **Number of studies** |  | **Test of association** |  |  | **Test of heterogeneity** |  | **Publication bias** |
| --- | --- | --- | --- | --- | --- | --- | --- | --- | --- |
|  |  |  | **OR** | **95% CI** | ***P*-value** | **Model** | ***P*-val** | **I^2^** | ***P*-value (Egger's test)** |
| **Allele contrast (A vs. C)** | | | | | | | | | |
|  | Asian | 16 | 1.17 | 1.1097-1.2283 | 2.3E-09 | Random | 0.08 | 0.3531 | 0.9874 |
|  | Non-Asians | 9 | 1.08 | 0.9762-1.1905 | 0.1378 | Random | 0 | 0.8176 | 0.9061 |
| **Recessive model (AA vs. AC+CC)** | | | | | | | | | |
|  | Asian | 16 | 1.25 | 1.1287-1.3841 | 1.81E-05 | Fixed | 0.45 | 0 | 0.7441 |
|  | Non-Asians | 9 | 1.15 | 0.9706-1.3517 | 0.108186 | Random | 0 | 0.7618 | 0.7295 |
| **Dominant model (AA+AC vs. CC)** | | | | | | | | | |
|  | Asian | 16 | 1.19 | 1.1169-1.2680 | 7.63E-08 | Random | 0.06 | 0.3883 | 0.6023 |
|  | Non-Asians | 9 | 1.09 | 0.9614-1.2384 | 0.177045 | Random | 0 | 0.7677 | 0.8901 |
| **Overdominant (AC vs. AA+CC)** | | | | | | | | | |
|  | Asian | 16 | 1.13 | 1.0504-1.2200 | 0.001163 | Random | 0.004 | 0.5442 | 0.2965 |
|  | Non-Asians | 9 | 1.03 | 0.9742-1.0844 | 0.315597 | Fixed | 0.33 | 0.1253 | 0.6668 |
| **pairw1 (AA vs. CC)** | | | | | | | | | |
|  | Asian | 16 | 1.31 | 1.1681-1.4505 | 1.83E-06 | Fixed | 0.35 | 0.0934 | 0.8393 |
|  | Non-Asians | 9 | 1.20 | 0.9698-1.4903 | 0.092966 | Random | 0 | 0.8263 | 0.8381 |
| **pairw2 (AA vs. AC)** | | | | | | | | | |
|  | Asian | 16 | 1.16 | 1.0437-1.2932 | 0.006118 | Fixed | 0.43 | 0.0181 | 0.885 |
|  | Non-Asians | 9 | 1.11 | 0.9623-1.2791 | 0.152683 | Random | 0.005 | 0.6322 | 0.579 |
| **pairw3 (AC vs. CC)** | | | | | | | | | |
|  | Asian | 16 | 1.17 | 1.0910-1.2513 | 8.6E-06 | Random | 0.03 | 0.4307 | 0.3548 |
|  | Non-Asians | 9 | 1.07 | 0.9547-1.1901 | 0.256164 | Random | 0.003 | 0.6529 | 0.8261 |
