# Supplementary material for: Type 2 diabetes linked FTO gene variant rs8050136 is significantly associated with gravidity in gestational diabetes in a sample of Bangladeshi women: Meta-analysis and case-control study
Source: PLoS One. 2023 Nov 30;18(11):e0288318. doi: 10.1371/journal.pone.0288318 (PMC10688623; doi:10.1371/journal.pone.0288318)
Supplement: S6 Table — (DOCX) [file pone.0288318.s006.docx]

**S6 Table: Subgroup analysis based on country**

| **Model** | **Country** | **Number of studies** |  | **Test of association** |  |  | **Test of heterogeneity** |  | **Publication bias** |
| --- | --- | --- | --- | --- | --- | --- | --- | --- | --- |
|  |  |  | **OR** | **95% CI** | ***P*-value** | **Model** | ***P*-** **value** | **I^2** | ***P*-** **value (Egger's test)** |
| **Allele contrast (A vs. C)** | | | | | | | | | |
|  | Bosnia | 1 | 1.02 | 0.8384-1.2404 | 0.84 | Fixed | NA | NA | NA |
|  | Brazil | 1 | 0.97 | 0.7295-1.2871 | 0.83 | Fixed | NA | NA | NA |
|  | China | 5 | 1.22 | 1.1382-1.2988 | 6.5E-09 | Fixed | 0.911 | 0 | 0.2399 |
|  | Finland | 1 | 0.9 | 0.8298-0.9785 | 0.013 | Fixed | NA | NA | NA |
|  | Hong Kong | 1 | 1.17 | 1.0574-1.2916 | 0.002 | Fixed | NA | NA | NA |
|  | India | 2 | 1.18 | 0.9383-1.4958 | 0.15 | Random | 0.047 | 0.75 | NA |
|  | Indonesia | 1 | 2.28 | 1.3138-3.9522 | 0.003 | Fixed | NA | NA | NA |
|  | Japan | 3 | 1.15 | 1.0533-1.2629 | 0.002 | Fixed | 0.479 | 0 | 0.6319 |
|  | Kazakhstan | 1 | 1.19 | 0.9845-1.4420 | 0.07 | Fixed | NA | NA | NA |
|  | Korea | 1 | 0.91 | 0.7250-1.1402 | 0.41 | Fixed | NA | NA | NA |
|  | Lebanon | 1 | 1.16 | 1.0291-1.3141 | 0.015 | Fixed | NA | NA | NA |
|  | Netherlands | 1 | 1.11 | 0.9927-1.2499 | 0.066 | Fixed | NA | NA | NA |
|  | Palestine | 1 | 0.76 | 0.5099-1.1190 | 0.162 | Fixed | NA | NA | NA |
|  | Sweden | 1 | 1.24 | 0.9348-1.6372 | 0.137 | Fixed | NA | NA | NA |
|  | UK | 1 | 1.26 | 1.1638-1.3721 | 2.53E-08 | Fixed | NA | NA | NA |
|  | USA | 3 | 1.08 | 0.9387-1.2509 | 0.27283 | Random | 0.01 | 0.779 | 0.6681 |
| **Recessive model (AA vs. AC+CC)** | | | | | | | | | |
|  | Bosnia | 1 | 0.93 | 0.6780-1.2803 | 0.66 | Fixed | NA | NA | NA |
|  | Brazil | 1 | 1.04 | 0.6166-1.7394 | 0.8947 | Fixed | NA | NA | NA |
|  | China | 5 | 1.35 | 1.1072-1.6435 | 0.00297 | Fixed | 0.95 | 0 | 0.0971 |
|  | Finland | 1 | 0.81 | 0.6922-0.9480 | 0.00865 | Fixed | NA | NA | NA |
|  | Hong Kong | 1 | 1.23 | 0.8365-1.8222 | 0.28866 | Fixed | NA | NA | NA |
|  | India | 2 | 1.11 | 0.8672-1.4293 | 0.39984 | Fixed | 0.433 | 0 | NA |
|  | Indonesia | 1 | 2.97 | 1.2245-7.1925 | 0.01602 | Fixed | NA | NA | NA |
|  | Japan | 3 | 1.32 | 1.0171-1.7251 | 0.03703 | Fixed | 0.29 | 0.192 | 0.876 |
|  | Kazakhstan | 1 | 0.87 | 0.5428-1.4006 | 0.57082 | Fixed | NA | NA | NA |
|  | Korea | 1 | 0.65 | 0.3002-1.4257 | 0.2857 | Fixed | NA | NA | NA |
|  | Lebanon | 1 | 1.33 | 1.0800-1.6297 | 0.0071 | Fixed | NA | NA | NA |
|  | Netherlands | 1 | 1.32 | 1.0649-1.6250 | 0.011 | Fixed | NA | NA | NA |
|  | Palestine | 1 | 0.95 | 0.5069-1.7803 | 0.8727 | Fixed | NA | NA | NA |
|  | Sweden | 1 | 1.30 | 0.7849-2.1383 | 0.31126 | Fixed | NA | NA | NA |
|  | UK | 1 | 1.31 | 1.1268-1.5210 | 0.00043 | Fixed | NA | NA | NA |
|  | USA | 3 | 1.26 | 0.9462-1.6723 | 0.11432 | Random | 0.02 | 0.739 | 0.7453 |
| **Dominant model (AA+AC vs. CC)** | | | | | | | | | |
|  | Bosnia | 1 | 1.13 | 0.8236-1.5414 | 0.4555 | Fixed | NA | NA | NA |
|  | Brazil | 1 | 0.92 | 0.6172-1.3722 | 0.6836 | Fixed | NA | NA | NA |
|  | China | 5 | 1.24 | 1.1509-1.3411 | 2.69E-08 | Fixed | 0.899 | 0 | 0.1322 |
|  | Finland | 1 | 0.91 | 0.8085-1.0238 | 0.12 | Fixed | NA | NA | NA |
|  | Hong Kong | 1 | 1.19 | 1.0656-1.3301 | 0.002 | Fixed | NA | NA | NA |
|  | India | 2 | 1.22 | 0.9529-1.5620 | 0.115 | Random | 0.076 | 0.683 | NA |
|  | Indonesia | 1 | 2.23 | 1.0056-4.9653 | 0.04843 | Fixed | NA | NA | NA |
|  | Japan | 3 | 1.16 | 1.0445-1.2945 | 0.00586 | Fixed | 0.10 | 0.562 | 0.6297 |
|  | Kazakhstan | 1 | 1.37 | 1.0785-1.7571 | 0.01026 | Fixed | NA | NA | NA |
|  | Korea | 1 | 0.93 | 0.7206-1.1951 | 0.56252 | Fixed | NA | NA | NA |
|  | Lebanon | 1 | 1.13 | 0.9347-1.3652 | 0.20736 | Fixed | NA | NA | NA |
|  | Netherlands | 1 | 1.06 | 0.9029-1.2524 | 0.46159 | Fixed | NA | NA | NA |
|  | Palestine | 1 | 0.52 | 0.2799-0.9802 | 0.04313 | Fixed | NA | NA | NA |
|  | Sweden | 1 | 1.34 | 0.8790-2.0381 | 0.1742 | Fixed | NA | NA | NA |
|  | UK | 1 | 1.41 | 1.2451-1.5968 | 6.11E-08 | Fixed | NA | NA | NA |
|  | USA | 3 | 1.06 | 0.8931-1.2657 | 0.49089 | Random | 0.026 | 0.726 | 0.0449 |
| **Overdominant (AC vs. AA+CC)** | | | | | | | | | |
|  | Bosnia | 1 | 1.16 | 0.8772-1.5279 | 0.30085 | Fixed | NA | NA | NA |
|  | Brazil | 1 | 0.90 | 0.6087-1.3411 | 0.61454 | Fixed | NA | NA | NA |
|  | China | 5 | 1.20 | 1.1057-1.2924 | 7.33E-06 | Fixed | 0.655 | 0 | 0.0899 |
|  | Finland | 1 | 1.02 | 0.9129-1.1468 | 0.69332 | Fixed | NA | NA | NA |
|  | Hong Kong | 1 | 1.18 | 1.0523-1.3199 | 0.00449 | Fixed | NA | NA | NA |
|  | India | 2 | 1.19 | 0.9142-1.5457 | 0.19685 | Random | 0.062 | 0.712 | NA |
|  | Indonesia | 1 | 0.89 | 0.3961-2.0008 | 0.77839 | Fixed | NA | NA | NA |
|  | Japan | 3 | 1.13 | 0.9014-1.4164 | 0.28931 | Random | 0.017 | 0.754 | 0.6524 |
|  | Kazakhstan | 1 | 1.45 | 1.1289-1.8497 | 0.00348 | Fixed | NA | NA | NA |
|  | Korea | 1 | 0.97 | 0.7457-1.2557 | 0.80451 | Fixed | NA | NA | NA |
|  | Lebanon | 1 | 0.91 | 0.7626-1.0771 | 0.26428 | Fixed | NA | NA | NA |
|  | Netherlands | 1 | 0.92 | 0.7803-1.0751 | 0.28269 | Fixed | NA | NA | NA |
|  | Palestine | 1 | 0.61 | 0.3504-1.0769 | 0.08889 | Fixed | NA | NA | NA |
|  | Sweden | 1 | 1.12 | 0.7539-1.6533 | 0.582 | Fixed | NA | NA | NA |
|  | UK | 1 | 1.15 | 1.0287-1.2953 | 0.01466 | Fixed | NA | NA | NA |
|  | USA | 3 | 0.99 | 0.9056-1.0783 | 0.7893 | Fixed | 0.55 | 0 | 0.5603 |
| **pairw1 (AA vs. CC)** | | | | | | | | | |
|  | Bosnia | 1 | 1.04 | 0.7055-1.5202 | 0.85813 | Fixed | NA | NA | NA |
|  | Brazil | 1 | 0.98 | 0.5567-1.7223 | 0.94182 | Fixed | NA | NA | NA |
|  | China | 5 | 1.44 | 1.1778-1.7648 | 0.00039 | Fixed | 0.972 | 0 | 0.1369 |
|  | Finland | 1 | 0.79 | 0.6638-0.9369 | 0.0069 | Fixed | NA | NA | NA |
|  | Hong Kong | 1 | 1.29 | 0.8702-1.8993 | 0.207 | Fixed | NA | NA | NA |
|  | India | 2 | 1.16 | 0.8937-1.5143 | 0.26089 | Fixed | 0.389 | 0 | NA |
|  | Indonesia | 1 | 3.60 | 1.3414-9.6615 | 0.011 | Fixed | NA | NA | NA |
|  | Japan | 3 | 1.39 | 1.0643-1.8152 | 0.01563 | Fixed | 0.519 | 0 | 0.9003 |
|  | Kazakhstan | 1 | 1.03 | 0.6310-1.6758 | 0.91073 | Fixed | NA | NA | NA |
|  | Korea | 1 | 0.65 | 0.2964-1.4146 | 0.2757 | Fixed | NA | NA | NA |
|  | Lebanon | 1 | 1.36 | 1.0653-1.7244 | 0.01334 | Fixed | NA | NA | NA |
|  | Netherlands | 1 | 1.31 | 1.0379-1.6481 | 0.0229 | Fixed | NA | NA | NA |
|  | Palestine | 1 | 0.61 | 0.2837-1.2915 | 0.19417 | Fixed | NA | NA | NA |
|  | Sweden | 1 | 1.50 | 0.8486-2.6462 | 0.16331 | Fixed | NA | NA | NA |
|  | UK | 1 | 1.57 | 1.3278-1.8670 | 1.78E-07 | Fixed | NA | NA | NA |
|  | USA | 3 | 1.29 | 0.8859-1.8804 | 0.1838 | Random | 0.004 | 0.821 | 0.859 |
| **pairw2 (****AA vs. AC)** | | | | | | | | | |
|  | Bosnia | 1 | 0.88 | 0.6260-1.2351 | 0.4582 | Fixed | NA | NA | NA |
|  | Brazil | 1 | 1.09 | 0.6241-1.9061 | 0.76054 | Fixed | NA | NA | NA |
|  | China | 5 | 1.20 | 0.9758-1.4733 | 0.08427 | Fixed | 0.941 | 0 | 0.3019 |
|  | Finland | 1 | 0.83 | 0.7002-0.9769 | 0.02540 | Fixed | NA | NA | NA |
|  | Hong Kong | 1 | 1.09 | 0.7284-1.6171 | 0.6873 | Fixed | NA | NA | NA |
|  | India | 2 | 1.05 | 0.8081-1.3699 | 0.70557 | Fixed | 0.787 | 0 | NA |
|  | Indonesia | 1 | 2.38 | 0.8657-6.5156 | 0.093 | Fixed | NA | NA | NA |
|  | Japan | 3 | 1.20 | 0.7773-1.857 | 0.40872 | Random | 0.089 | 0.59 | 0.8403 |
|  | Kazakhstan | 1 | 0.71 | 0.4329-1.1618 | 0.17240 | Fixed | NA | NA | NA |
|  | Korea | 1 | 0.68 | 0.3028-1.5124 | 0.34118 | Fixed | NA | NA | NA |
|  | Lebanon | 1 | 1.31 | 1.0511-1.6310 | 0.01620 | Fixed | NA | NA | NA |
|  | Netherlands | 1 | 1.32 | 1.0540-1.6581 | 0.01572 | Fixed | NA | NA | NA |
|  | Palestine | 1 | 1.26 | 0.6373-2.4883 | 0.507 | Fixed | NA | NA | NA |
|  | Sweden | 1 | 1.17 | 0.6862-1.9874 | 0.5675 | Fixed | NA | NA | NA |
|  | UK | 1 | 1.16 | 0.9913-1.3605 | 0.06409 | Fixed | NA | NA | NA |
|  | USA | 3 | 1.20 | 1.0454-1.3662 | 0.00905 | Fixed | 0.112 | 0.542 | 0.6053 |
| **pairw3 (AC vs. CC)** | | | | | | | | | |
|  | Bosnia | 1 | 1.18 | 0.8425-1.6464 | 0.33844 | Fixed | NA | NA | NA |
|  | Brazil | 1 | 0.9 | 0.5840-1.3803 | 0.62316 | Fixed | NA | NA | NA |
|  | China | 5 | 1.22 | 1.1287-1.3218 | 6.87E-07 | Fixed | 0.827 | 0 | 0.0887 |
|  | Finland | 1 | 0.95 | 0.8415-1.0805 | 0.45547 | Fixed | NA | NA | NA |
|  | Hong Kong | 1 | 1.18 | 1.0574-1.3270 | 0.00347 | Fixed | NA | NA | NA |
|  | India | 2 | 1.21 | 0.9458-1.5487 | 0.12928 | Random | 0.088 | 0.656 | NA |
|  | Indonesia | 1 | 1.51 | 0.6060-3.7917 | 0.37393 | Fixed | NA | NA | NA |
|  | Japan | 3 | 1.15 | 0.9301-1.4250 | 0.19574 | Random | 0.029 | 0.718 | 0.6425 |
|  | Kazakhstan | 1 | 1.45 | 1.1244-1.8699 | 0.00418 | Fixed | NA | NA | NA |
|  | Korea | 1 | 0.96 | 0.7369-1.2429 | 0.74175 | Fixed | NA | NA | NA |
|  | Lebanon | 1 | 1.04 | 0.8455-1.2674 | 0.73787 | Fixed | NA | NA | NA |
|  | Netherlands | 1 | 0.99 | 0.8301-1.1790 | 0.90415 | Fixed | NA | NA | NA |
|  | Palestine | 1 | 0.48 | 0.2443-0.9458 | 0.0339 | Fixed | NA | NA | NA |
|  | Sweden | 1 | 1.28 | 0.8214-2.004 | 0.27329 | Fixed | NA | NA | NA |
|  | UK | 1 | 1.36 | 1.1891-1.5459 | 5.46E-06 | Fixed | NA | NA | NA |
|  | USA | 3 | 1.04 | 0.9433-1.1413 | 0.4479 | Fixed | 0.103 | 0.559 | 0.06 |
