# Supplementary material for: Type 2 diabetes linked FTO gene variant rs8050136 is significantly associated with gravidity in gestational diabetes in a sample of Bangladeshi women: Meta-analysis and case-control study
Source: PLoS One. 2023 Nov 30;18(11):e0288318. doi: 10.1371/journal.pone.0288318 (PMC10688623; doi:10.1371/journal.pone.0288318)
Supplement: S7 Table — (DOCX) [file pone.0288318.s007.docx]

**S7 Table: General characteristics for primigravida and multigravida women**

| **Primigravida (208)** | **Control (129)** | **GDM (79)** | ***P* value** |
| --- | --- | --- | --- |
| Age | 23.72±4.17 | 25.81±4.57 | 0.0009 |
| BMI | 24.74±3.92 | 25.82±3.47 | 0.0454 |
| SBP | 107.64±12.52 | 108.21±11.25 | 0.7410 |
| DBP | 67.91±8.24 | 69.74±8.79 | 0.1312 |
| FPG | 4.30±0.47 | 5.06±0.68 | < 0.0001 |
| OPG | 7.48±1.19 | 9.76±1.65 | < 0.0001 |
| TPG | 6.21±1.05 | 8.22±1.51 | < 0.0001 |
| FHO yes | 43(33%) | 41(52%) | 0.0068 |
| **Multigravida (289)** | **Control (152)** | **GDM (137)** |  |
| Age | 26.91±4.80 | 28.58±4.33 | 0.0022 |
| BMI | 25.47±3.71 | 27.12±4.26 | 0.0005 |
| SBP | 109.57±11.34 | 109.78±12.11 | 0.8791 |
| DBP | 69.47±9.42 | 71.11±9.36 | 0.1394 |
| FPG | 4.34±0.47 | 5.20±0.69 | < 0.0001 |
| OPG | 7.51±1.25 | 9.95±1.55 | < 0.0001 |
| TPG | 6.61±1.02 | 8.27±1.48 | < 0.0001 |
| FHO yes | 48(31%) | 59(43%) | 0.0349 |
|  | **Primigravida (208)** | **Multigravida (289)** |  |
| Age | 24.51±4.44 | 27.70±4.65 | < 0.0001 |
| BMI | 25.15±3.78 | 26.23±4.05 | 0.0027 |
| SBP | 107.85±12.03 | 109.67±11.69 | 0.0914 |
| DBP | 68.59±8.47 | 70.24±9.41 | 0.0450 |
| FPG | 4.59±0.67 | 4.75±0.73 | 0.0130 |
| OPG | 8.35±1.77 | 8.67±1.86 | 0.0541 |
| TPG | 6.98± 1.58 | 7.39±1.51 | 0.0036 |
| FHO yes | 84(40%) | 107(37%) | 0.4977 |
| Control | 129(62%) | 152(52%) | 0.0268 |
| GDM | 79(37%) | 137(47%) | 0.03 |
| **BMI ≥30** | **Control (30)** | **GDM (34)** | ***P* value** |
| Age | 26.73±5.15 | 28.24±3.74 | 0.18 |
| BMI | 32.45±1.79 | 32.68±2.3 | 0.66 |
| SBP | 112±11.26 | 115.88±8.92 | 0.13 |
| DBP | 73.67±9.9 | 76.32±9.15 | 0.27 |
| FPG | 4.36±0.49 | 5.28±0.60 | < 0.0001 |
| OPG | 7.58±1.09 | 10.52±1.35 | < 0.0001 |
| TPG | 6.66±0.92 | 8.59±1.34 | < 0.0001 |
| FHO yes | 21(70%) | 15(44%) | 0.04 |
