# Supplementary material for: Type 2 diabetes linked FTO gene variant rs8050136 is significantly associated with gravidity in gestational diabetes in a sample of Bangladeshi women: Meta-analysis and case-control study
Source: PLoS One. 2023 Nov 30;18(11):e0288318. doi: 10.1371/journal.pone.0288318 (PMC10688623; doi:10.1371/journal.pone.0288318)
Supplement: S8 Table — a adjusted for gravidity and family history of diabetes. (DOCX) [file pone.0288318.s008.docx]

**S8 Table: Association of rs8050136 with GDM under different genetic model**

| **Model** | **Control (%)** | **GDM (%)** | **OR (95% CI)** | ***P* value** | **OR (95% CI) ^a^** | ***P* value ^a^** |
| --- | --- | --- | --- | --- | --- | --- |
| **Codominant**  **C/C**  **A/C**  **A/A** | 143 (50.4%) | 101(46.3%) | 1.00 | 0.28 | 1.00 | 0.37 |
|  | 126 (44.4%) | 98 (45%) | 1.10 (0.76-1.59) |  | 1.09 (0.75-1.58) |  |
|  | 15 (5.3%) | 19 (8.7%) | 1.79 (0.87-3.70) |  | 1.70 (0.81-3.55) |  |
| **Dominant**  **C/C**  **A/C-A/A** | 143 (50.4%) | 101(46.3%) | 1.00 | 0.37 | 1.00 | 0.44 |
|  | 141 (49.6%) | 117(53.7%) | 1.17 (0.82-1.67) |  | 1.15 (0.81-1.65) |  |
| **Recessive**  **C/C-A/C**  **A/A** | 269 (94.7%) | 199(91.3%) | 1.00 | 0.13 | 1.00 | 0.18 |
|  | 15 (5.3%) | 19 (8.7%) | 1.71 (0.85-3.45) |  | 1.63 (0.80-3.33) |  |
| **Overdominant**  **C/C-A/A**  **A/C** | 158 (55.6%) | 120(55%) | 1.00 | 0.9 | 1.00 | 0.92 |
|  | 126 (44.4%) | 98 (45%) | 1.02 (0.72-1.46) |  | 1.02 (0.71-1.46) |  |
| **Log-additive** | --- | --- | 1.22 (0.91-1.62) | 0.18 | 1.19 (0.89-1.60) | 0.24 |

**^a^ adjusted for gravidity and family history of diabetes**
