# Supplementary material for: Type 2 diabetes linked FTO gene variant rs8050136 is significantly associated with gravidity in gestational diabetes in a sample of Bangladeshi women: Meta-analysis and case-control study
Source: PLoS One. 2023 Nov 30;18(11):e0288318. doi: 10.1371/journal.pone.0288318 (PMC10688623; doi:10.1371/journal.pone.0288318)
Supplement: S9 Table — a adjusted for gravidity. (DOCX) [file pone.0288318.s009.docx]

**S9 Table: Cross classification interaction table of *FTO* variant rs8050136 and family history of T2DM under different genetic model**

| **Models** | **Family history of T2DM (n=502)** | | | | | | **Interaction**  ***P* value ^a^** |
| --- | --- | --- | --- | --- | --- | --- | --- |
|  | **No** | | | **Yes** | | |  |
|  | **Control** | **GDM** | **OR**  **(95% CI)** | **Control** | **GDM** | **OR**  **(95% CI)** |  |
| **Codominant**  **C/C**  **A/C**  **A/A** | 102 | 54 | 1.00 | 41 | 47 | **2.22**  **(1.30-3.80)** | 0.55 |
|  | 82 | 54 | 1.25  (0.77-2.01) | 44 | 44 | **1.95**  **(1.14-3.34)** |  |
|  | 8 | 9 | 2.26  (0.82-6.24) | 7 | 10 | 2.67  (0.96-7.44) |  |
| **Dominant**  **C/C**  **A/C-A/A** | 102 | 54 | 1.00 | 41 | 47 | **2.22**  **(1.30-3.79)** | 0.33 |
|  | 90 | 63 | 1.33  (0.84-2.12) | 51 | 54 | **2.05**  **(1.23-3.41)** |  |
| **Recessive**  **C/C-A/C**  **A/A** | 184 | 108 | 1.00 | 85 | 91 | **1.88**  **(1.28-2.75)** | 0.52 |
|  | 8 | 9 | 2.04  (0.76-5.47) | 7 | 10 | 2.40  (0.88-6.53) |  |
| **Overdominant**  **C/C-A/A**  **A/C** | 110 | 63 | 1.00 | 48 | 57 | **2.10**  **(1.28-3.45)** | 0.43 |
|  | 82 | 54 | 1.15  (0.72-1.82) | 44 | 44 | **1.79**  **(1.06-3.03)** |  |

**^a^ adjusted for gravidity**
