# Supplementary material for: Type 2 diabetes linked FTO gene variant rs8050136 is significantly associated with gravidity in gestational diabetes in a sample of Bangladeshi women: Meta-analysis and case-control study
Source: PLoS One. 2023 Nov 30;18(11):e0288318. doi: 10.1371/journal.pone.0288318 (PMC10688623; doi:10.1371/journal.pone.0288318)
Supplement: S10 Table — a adjusted for family history of diabetes. (DOCX) [file pone.0288318.s010.docx]

**S10 Table:** **Cross classification interaction table of *FTO* variant rs8050136 and gravidity under**

**different genetic models**

| **Models** | **Gravidity(n=502)** | | | | | | **Interaction**  ***P* value ^a^** |
| --- | --- | --- | --- | --- | --- | --- | --- |
|  | **Primigravida** | | | **Multigravida** | | |  |
|  | **Control** | **GDM** | **OR**  **(95% CI)** | **Control** | **GDM** | **OR**  **(95% CI)** |  |
| **Codominant**  **C/C**  **A/C**  **A/A** | 57 | 45 | 1.00 | 86 | 56 | 0.84  (0.50-1.42) | **0.0068** |
|  | 67 | 29 | 0.53  (0.29-0.95) | 59 | 69 | 1.51  (0.89-2.56) |  |
|  | 8 | 7 | 1.10  (0.37-3.30) | 7 | 12 | 1.96  (0.70-5.44) |  |
| **Dominant**  **C/C**  **A/C-A/A** | 57 | 45 | 1.00 | 86 | 56 | 0.84  (0.50-1.42) | **0.0021** |
|  | 75 | 36 | 0.59  (0.33-1.03) | 66 | 81 | 1.56  (0.93-2.60) |  |
| **Recessive**  **C/C-A/C**  **A/A** | 124 | 74 | 1.00 | 145 | 125 | 1.50  (1.03-2.19) | 0.81 |
|  | 8 | 7 | 1.48  (0.51-4.30) | 7 | 12 | 2.64  (0.99-7.09) |  |
| **Overdominant**  **C/C-A/A**  **A/C** | 65 | 52 | 1.00 | 93 | 68 | 0.92  (0.57-1.49) | **0.0025** |
|  | 67 | 29 | 0.52  (0.29-0.92) | 59 | 69 | 1.49  (0.90-2.48) |  |

**^a^ adjusted for family history of diabetes**
