# Supplementary material for: Type 2 diabetes linked FTO gene variant rs8050136 is significantly associated with gravidity in gestational diabetes in a sample of Bangladeshi women: Meta-analysis and case-control study
Source: PLoS One. 2023 Nov 30;18(11):e0288318. doi: 10.1371/journal.pone.0288318 (PMC10688623; doi:10.1371/journal.pone.0288318)
Supplement: S11 Table — a adjusted for family history of diabetes. (DOCX) [file pone.0288318.s011.docx]

**S11 Table:** **Association of rs8050136 with GDM under different genetic models in primigravida women; N=208**

| **Model** | **Control (%)** | **GDM (%)** | **OR (95% CI)** | ***P* value** | **OR (95% CI) ^a^** | ***P* value ^a^** |
| --- | --- | --- | --- | --- | --- | --- |
| **Codominant**  C/C  A/C  A/A | 56 (43.4%) | 44 (55.7%) | 1.00 | 0.1 | 1.00 | 0.072 |
|  | 65 (50.4%) | 28 (35.4%) | 0.55  (0.30-0.99) |  | 0.51  (0.28-0.94) |  |
|  | 8 (6.2%) | 7 (8.9%) | 1.11  (0.37-3.31) |  | 1.10  (0.36-3.34) |  |
| **Dominant**  C/C  A/C-A/A | 56 (43.4%) | 44 (55.7%) | 1.00 | 0.085 | 1.00 | 0.06 |
|  | 73 (56.6%) | 35 (44.3%) | 0.61  (0.35-1.07) |  | 0.58  (0.32-1.03) |  |
| **Recessive**  C/C-A/C  A/A | 121 (93.8%) | 72 (91.1%) | 1.00 | 0.48 | 1.00 | 0.47 |
|  | 8 (6.2%) | 7 (8.9%) | 1.47  (0.51-4.23) |  | 1.50  (0.51-4.38) |  |
| **Overdominant**  C/C-A/A  A/C | 64 (49.6%) | 51 (64.6%) | 1.00 | **0.034** | 1.00 | **0.022** |
|  | 65 (50.4%) | 28 (35.4%) | 0.54  (0.30-0.96) |  | 0.51  (0.28-0.91) |  |
| **Log-additive** | --- | --- | 0.78  (0.49-1.23) | 0.28 | 0.75  (0.47-1.20) | 0.23 |

**^a^ adjusted for family history of diabetes**
