# Supplementary material for: Type 2 diabetes linked FTO gene variant rs8050136 is significantly associated with gravidity in gestational diabetes in a sample of Bangladeshi women: Meta-analysis and case-control study
Source: PLoS One. 2023 Nov 30;18(11):e0288318. doi: 10.1371/journal.pone.0288318 (PMC10688623; doi:10.1371/journal.pone.0288318)
Supplement: S12 Table — a adjusted for family history of diabetes. (DOCX) [file pone.0288318.s012.docx]

**S12 Table: Association of rs8050136 with GDM under different genetic models in multigravida women; N=289**

| **Model** | **Control (%)** | **GDM (%)** | **OR (95% CI)** | ***P* value** | **OR (95% CI) ^a^** | ***P* value ^a^** |
| --- | --- | --- | --- | --- | --- | --- |
| **Codominant**  C/C  A/C  A/A | 86 (56.6%) | 56 (40.9%) | 1.00 | **0.021** | 1.00 | **0.025** |
|  | 59 (38.8%) | 69 (50.4%) | **1.80 (1.11-2.91)** |  | **1.83 (1.12-2.97)** |  |
|  | 7 (4.6%) | 12 (8.8%) | 2.63 (0.98-7.09) |  | 2.43 (0.89-6.62) |  |
| **Dominant**  C/C  A/C-A/A | 86 (56.6%) | 56 (40.9%) | 1.00 | **0.0075** | 1.00 | **0.0078** |
|  | 66 (43.4%) | 81 (59.1%) | **1.88 (1.18-3.01)** |  | **1.89 (1.18-3.03)** |  |
| **Recessive**  C/C-A/C  A/A | 145 (95.4%) | 125 (91.2%) | 1.00 | 0.15 | 1.00 | 0.22 |
|  | 7 (4.6%) | 12 (8.8%) | 1.99 (0.76-5.21) |  | 1.82 (0.69-4.81) |  |
| **Overdominant**  C/C-A/A  A/C | 93 (61.2%) | 68 (49.6%) | 1.00 | **0.048** | 1.00 | **0.039** |
|  | 59 (38.8%) | 69 (50.4%) | **1.60 (1.00-2.55)** |  | **1.64 (1.02-2.63)** |  |
| **Log-additive** | --- | --- | **1.71 (1.16-2.52)** | **0.0058** | **1.69 (1.14-2.51)** | **0.0075** |

**^a^ adjusted for family history of diabetes**
