# Supplementary material for: Type 2 diabetes linked FTO gene variant rs8050136 is significantly associated with gravidity in gestational diabetes in a sample of Bangladeshi women: Meta-analysis and case-control study
Source: PLoS One. 2023 Nov 30;18(11):e0288318. doi: 10.1371/journal.pone.0288318 (PMC10688623; doi:10.1371/journal.pone.0288318)
Supplement: S13 Table — (DOCX) [file pone.0288318.s013.docx]

**S13 Table: Cross classification interaction table of *FTO* variant rs8050136 and family history of T2DM under different genetic models in primigravida group**

| **Models** | **Family history of T2DM (n=208)** | | | | | | **Interaction**  ***P* value** |
| --- | --- | --- | --- | --- | --- | --- | --- |
|  | **No** | | | **Yes** | | |  |
|  | **Control** | **GDM** | **OR (95% CI)** | **Control** | **GDM** | **OR (95% CI)** |  |
| **Codominant**  **C/C**  **A/C**  **A/A** | 40 | 22 | 1.00 | 16 | 22 | 2.50  (1.09-5.72) | 0.83 |
|  | 41 | 12 | 0.53  (0.23-1.22) | 24 | 16 | 1.21  (0.53-2.75) |  |
|  | 5 | 4 | 1.45  (0.35-5.98) | 3 | 3 | 1.82  (0.34-9.78) |  |
| **Dominant**  **C/C**  **A/C-A/A** | 40 | 22 | 1.00 | 16 | 22 | 2.50  (1.09-5.72) | 0.72 |
|  | 46 | 16 | 0.63  (0.29-1.37) | 27 | 19 | 1.28  (0.58-2.80) |  |
| **Recessive**  **C/C-A/C**  **A/A** | 81 | 34 | 1.00 | 40 | 38 | 2.26  (1.24-4.12) | 0.59 |
|  | 5 | 4 | 1.91  (0.48-7.53) | 3 | 3 | 2.38  (0.46-12.40) |  |
| **Overdominant**  **C/C-A/A**  **A/C** | 45 | 26 | 1.00 | 19 | 25 | 2.28  (1.06-4.91) | 1 |
|  | 41 | 12 | 0.51  (0.23-1.13) | 24 | 16 | 1.15  (0.52-2.56) |  |
