# Supplementary material for: Type 2 diabetes linked FTO gene variant rs8050136 is significantly associated with gravidity in gestational diabetes in a sample of Bangladeshi women: Meta-analysis and case-control study
Source: PLoS One. 2023 Nov 30;18(11):e0288318. doi: 10.1371/journal.pone.0288318 (PMC10688623; doi:10.1371/journal.pone.0288318)
Supplement: S14 Table — (DOCX) [file pone.0288318.s014.docx]

**S14 Table: Cross classification interaction table of *FTO* variant rs8050136 and family history of T2DM under different genetic model in multigravida group**

| **Models** | **Family history of T2DM (n=288)** | | | | | | **Interaction**  ***P* value** |
| --- | --- | --- | --- | --- | --- | --- | --- |
|  | **No** | | | **Yes** | | |  |
|  | **Control** | **GDM** | **OR (95% CI)** | **Control** | **GDM** | **OR (95% CI)** |  |
| **Codominant**  **C/C**  **A/C**  **A/A** | 61 | 31 | 1.00 | 25 | 24 | 1.89 (0.93-3.83) | 0.78 |
|  | 40 | 41 | 2.02  (1.09-3.73) | 19 | 28 | 2.90  (1.40-5.99) |  |
|  | 3 | 5 | 3.28  (0.74-14.63) | 4 | 7 | 3.44  (0.94-12.67) |  |
| **Dominant**  **C/C**  **A/C-A/A** | 61 | 31 | 1.00 | 25 | 24 | 1.89  (0.93-3.83) | 0.57 |
|  | 43 | 46 | 2.11  (1.16-3.83) | 23 | 35 | 2.99  (1.52-5.92) |  |
| **Recessive**  **C/C-A/C**  **A/A** | 101 | 72 | 1.00 | 44 | 52 | 1.66  (1.00-2.74) | 0.65 |
|  | 3 | 5 | 2.34  (0.54-10.10) | 4 | 7 | 2.45  (0.69-8.70) |  |
| **Overdominant**  **C/C-A/A**  **A/C** | 64 | 36 | 1.00 | 29 | 31 | 1.90  (0.99-3.64) | 0.58 |
|  | 40 | 41 | 1.82  (1.00-3.31) | 19 | 28 | 2.62  (1.29-5.34) |  |
