# Supplementary material for: Type 2 diabetes linked FTO gene variant rs8050136 is significantly associated with gravidity in gestational diabetes in a sample of Bangladeshi women: Meta-analysis and case-control study
Source: PLoS One. 2023 Nov 30;18(11):e0288318. doi: 10.1371/journal.pone.0288318 (PMC10688623; doi:10.1371/journal.pone.0288318)
Supplement: S15 Table — (DOCX) [file pone.0288318.s015.docx]

**S15 Table: Excluded papers that have been sought and assessed for eligibility and reasons for exclusion**

| **Paper** | **Title** | **Reason for exclusion** |
| --- | --- | --- |
| Knuppel et al., 2015 | Joint Effect of Unlinked Genotypes: Application to Type 2 Diabetes in the EPIC-Potsdam Case-Cohort Study | Not a case-control study |
| Bai et al., 2015 | Association Analysis of Genetic Variants with Type 2 Diabetes in a Mongolian Population in China | Did not provide the genotype frequency |
| Kawk et al., 2013 | Clinical and Genetic Risk Factors for Type 2 Diabetes at Early or Late Post Partum After Gestational Diabetes Mellitus | Did not investigate the aim of the review, i.e. association of rs8050136 with T2D |
| Lauenborg et al., 2009 | Common Type 2 Diabetes Risk Gene Variants Associate with Gestational Diabetes | Did not investigate the aim of the review, i.e. association of rs8050136 with T2D |
| Chikowore et al., 2015 | Common Variants Associated with Type 2 Diabetes in a Black South African Population of Setswana Descent: African Populations Diverge | Did not provide the genotype frequency |
| Gamboa-Meléndez et al., 2012 | Contribution of Common Genetic Variation to the Risk of Type 2 Diabetes in the Mexican Mestizo Population | Did not provide the genotype frequency |
| Abbas et al., 2013 | Association of Genetic polymorphism of PPARγ-2, ACE, MTHFR, FABP-2 and FTO genes in risk prediction of type 2 diabetes mellitus | Did not investigate the aim of the review, i.e. association of rs8050136 with T2D |
| Hanson et al., 2015 | Role of Established Type 2 Diabetes–Susceptibility Genetic Variants in a High Prevalence American Indian Population | Did not provide the genotype frequency |
| Kong et al., 2015 | The Association of Type 2 Diabetes Loci Identified in Genome-Wide Association Studies with Metabolic Syndrome and Its Components in a Chinese Population with Type 2 Diabetes | Did not investigate the aim of the review, i.e. association of rs8050136 with T2D |
| Ng et al., 2013 | Transferability and Fine Mapping of Type 2 Diabetes Loci in African Americans | Not a case-control study |
| Votsi et al., 2017 | Type 2 Diabetes Susceptibility in the Greek-Cypriot Population: Replication of Associations with TCF7L2, FTO, HHEX, SLC30A8 and IGF2BP2 Polymorphisms | Did not provide the genotype frequency |
| Cho et al., 2008 | Type 2 diabetes-associated genetic variants discovered in the recent genome-wide association studies are related to gestational diabetes mellitus in the Korean population | Did not investigate the aim of the review, i.e. association of rs8050136 with T2D |
| Sanghera et al., 2008 | Impact of nine common type 2 diabetes risk polymorphisms in Asian Indian Sikhs: PPARG2 (Pro12Ala), IGF2BP2, TCF7L2 and FTO variants confer a significant risk | Did not investigate the aim of the review, i.e. association of rs8050136 with T2D |
| Kong et al., 2016 | Genetic variants associated with lean and obese type 2 diabetes in a Han Chinese population | Did not investigate the aim of the review, i.e. association of rs8050136 with T2D |
| Song et al., 2008 | FTO Polymorphisms Are Associated With Obesity but Not Diabetes Risk in Postmenopausal Women | Did not follow HWE |
| Nikitin et al., 2017 | Association of polymorphic markers of genes FTO, KCNJ11, CDKAL1, SLC30A8, and CDKN2B with type 2 diabetes mellitus in the Russian population | Did not follow HWE |
| Yako et al., 2015 | Contribution of ENPP1, TCF7L2, and FTO polymorphisms to type 2 diabetes in mixed ancestry ethnic population of South Africa | Not a case-control study |
| Anghebem-Oliveira et al., 2017 | Type 2 diabetes-associated genetic variants of FTO, LEPR, PPARg, and TCF7L2 in gestational diabetes in a Brazilian population | Did not investigate the aim of the review, i.e. association of rs8050136 with T2D |
| Shabana et al., 2015 | Effect of six type II diabetes susceptibility loci and an FTO variant on obesity in Pakistani subjects | Did not provide the genotype frequency |
| Abou-Hussein et al., 2011 | Genetic factors in risk assessment for the development of type 2 diabetes mellitus in a small case series | Not a case-control study |
| Hertel et al., 2008 | Genetic analysis of recently identified type 2 diabetes loci in 1,638 unselected patients with type 2 diabetes and 1,858 control participants from a Norwegian population-based cohort (the HUNT study) | Did not provide the genotype frequency |
| Ng et al., 2010 | Implication of Genetic Variants Near NEGR1, SEC16B, TMEM18, ETV5/DGKG, GNPDA2, LIN7C/BDNF, MTCH2, BCDIN3D/FAIM2, SH2B1, FTO, MC4R, and KCTD15 with Obesity and Type 2 Diabetes in 7705 Chinese | Did not provide the genotype frequency |
