# Supplementary material for: Type 2 diabetes linked FTO gene variant rs8050136 is significantly associated with gravidity in gestational diabetes in a sample of Bangladeshi women: Meta-analysis and case-control study
Source: PLoS One. 2023 Nov 30;18(11):e0288318. doi: 10.1371/journal.pone.0288318 (PMC10688623; doi:10.1371/journal.pone.0288318)
Supplement: S1 File — (DOCX) [file pone.0288318.s016.docx]

**Meta-analysis on Genetic Association Studies Checklist | PLOS ONE**

|  | Item | Section name and paragraph number within manuscript |
| --- | --- | --- |
|  | **Introduction** |  |
| 1 | Provide a detailed justification for the polymorphism studied; if a single polymorphism was analyzed, give details as to why others were not included in the meta-analysis. | Introduction, 4^th^ paragraph |
| 2 | Provide a detailed justification for the population(s) and clinical condition studied. | Introduction,1^st^ to 5^th^ paragraph |
|  | **Methods** |  |
| 3 | Provide full details of the search strategy employed; outline the full electronic search strategy –specific combination of keywords and any limits applied- for at least one database. Specify whether synonyms of polymorphisms/genes (e.g. SNP number) were searched. | Methods, 2.1 Meta-analysis, 2.1.1 Data Retrieval Strategy, 1^st^ paragraph |
| 4 | Report full details on the inclusion and exclusion criteria applied for selecting studies.  *Please list the excluded articles and the reasons for exclusion of each article in a supplementary file.* | Methods, 2.1 Meta-analysis, 2.1.2. Inclusion and Exclusion Criteria, 1st paragraph, supplementary file 1, S15 Table |
| 5 | Provide details on how the quality of the studies included in the analyses was assessed. | Methods, 2.1 Meta-analysis, 2.1.4 Quality Assessment and HWE Conformance of the Studies, 1st paragraph |
| 6 | Describe steps taken to contact study authors to identify additional studies and to request missing data. | Not applicable |
| 7 | Describe how environmental effects were adjusted for, if this adjustment was not conducted, outline the reasons for this. | Not applicable |
| 8 | Describe the methods of handling heterogeneity/between-study variance. | Methods, 2.1 Meta-analysis, 2.1.5 Statistical analyses, 1st paragraph |
| 9 | Describe how the Hardy-Weinberg equilibrium and linkage disequilibrium were assessed. | Methods, 2.1 Meta-analysis, 2.1.4 Quality Assessment and HWE Conformance of the Studies, 1st paragraph |
| 10 | Describe and justify the choice of model for the analyses (per-allele vs per-genotype vs genetic model-free, random effects vs fixed effects). | Methods, 2.1 Meta-analysis, 2.1.5 Statistical analyses, 1st paragraph and Discussion 2nd paragraph |
| 11 | Describe whether a sensitivity analysis has been completed. | Methods Section 2.1.5, line no 189-191,  Results section 3.1.2, line 324-326, Figure 5 |
| 12 | Describe whether an assessment of the effects of population stratification has been conducted. | Discussion, 2^nd^ -7^th^ paragraph |
| 13 | Describe whether study-specific results have been assessed and if so the reasons for this (e.g. forest plot). | Figure 4 |
|  | **Results** |  |
| 14 | Include flow diagram for the studies included in the meta-analysis as the first figure for the manuscript | Figure 2 (our paper contains both meta-analysis and case-control study) |
| 15 | Report details on allele/genotype prevalence. | Table 1 |
| 16 | Report the effect size estimates and p values for each analysis. | Result,  3.1 Meta-analysis, 3.1.2 FTO rs8050136 polymorphism significantly increase T2DM risk in Asians |
|  | **Discussion** |  |
| 17 | Discuss the limitations of the meta-analysis, including genotyping errors/bias and publication bias. | S1 Table, Discussion 4^th^ paragraph |
| 18 | If the meta-analysis identifies an association within a subgroup of the population studied but not another, discuss the implications of these results, and if applicable the possibility of subgroup-specific publication bias. | S5 Table, S6 Table |
| 19 | Discuss the suitability of the sample size employed to the research question and the power of the study. | Discussion 5^th^ and 9^th^ paragraph |
