# Supplementary figures and images for: Type 2 diabetes linked FTO gene variant rs8050136 is significantly associated with gravidity in gestational diabetes in a sample of Bangladeshi women: Meta-analysis and case-control study
Source: PLoS One. 2023 Nov 30;18(11):e0288318. doi: 10.1371/journal.pone.0288318 (PMC10688623; doi:10.1371/journal.pone.0288318)

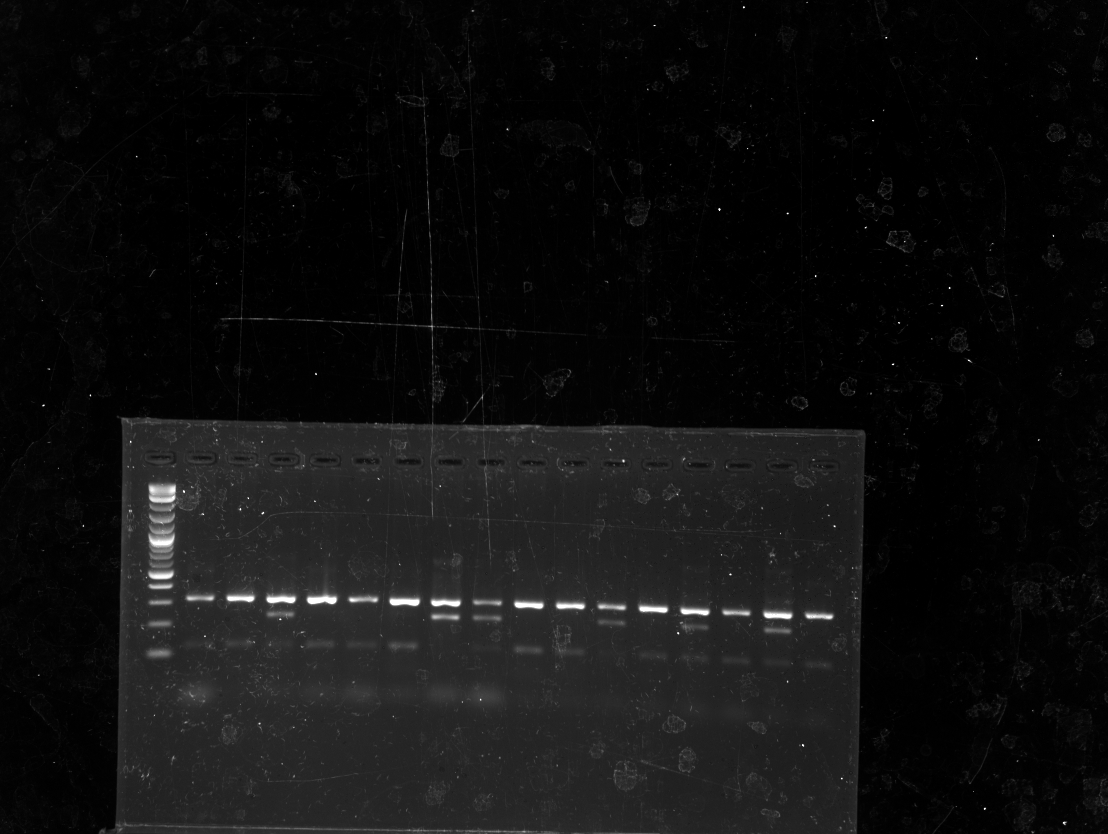

Supplement: S1 Fig — (PNG) [file pone.0288318.s018.png]
